# Armoured Amazon female^†^ moths: urticating setae in Notodontidae (Lepidoptera)

**DOI:** 10.1093/jisesa/ieag051

**Published:** 2026-07-02

**Authors:** Mizuki Uemura, Myron P Zalucki, Andrea Battisti

**Affiliations:** School of the Environment, The University of Queensland, St Lucia, QLD, Australia; School of the Environment, The University of Queensland, St Lucia, QLD, Australia; Department of Agronomy, Food, Natural Resources, Animals and Environment, University of Padova, Legnaro, Padova, Italy

**Keywords:** anal-tuft, hair, defense, lepidopterism, venomous

## Abstract

Urticating setae in the corethrogyne of female moths of Notodontidae (Anaphinae and Thaumetopoeinae) from Africa, Asia, and Australia were compared using light and scanning electron microscopy. In all 14 species examined, the proximal tip of the setae was spear-shaped with a lateral opening and had lobed or serrated barbs. Notable variation among genera was observed at the distal end of the seta, characterized by 5 non-exclusive forms: tapered (genera *Adrallia* Walker, *Anaphe* Walker, *Hypsoides* Butler, *Paradrallia* Bethune-Baker, and *Tanystola* Turner), spatulate (genera *Adrallia*, *Epanaphe* Aurivillius, *Epicoma* Hübner, *Hypsoides*, and *Trichiocercus* Stephens), blunt (genus *Paradrallia*), cup-shaped (genus *Ochrogaster* Herrich-Schäffer), or hook-shaped (genus *Gazalina* Walker). Verrucose projections on the distal end of the seta were present in 4 genera of Anaphinae (*Adrallia*, *Anaphe*, *Epanaphe*, and *Hypsoides*) and in 1 genus of Thaumetopoeinae (*Epicoma*), and their functional significance is discussed. Phylogenetic analyses of the cytochrome c oxidase subunit I gene region provided a framework for comparing setal morphology and geographic distribution among genera. This study is the first to compare and describe the morphology of urticating setae across females of multiple notodontid species. The shared setal morphologies, which appear to enhance aerodynamics and facilitate toxin dispersal, indicate that notodontid moths have evolved highly effective defense strategies and protection of their egg mass.

## Introduction

Among insects, Lepidoptera are well known to cause adverse reactions and medical complications to human and animal health. Much of the research on urticating Lepidoptera has focused on the medical complications in humans and domesticated animals from contact with larvae. A recent review by Battisti et al. (2024) provided an updated list of 576 species of venomous Lepidoptera from 14 families and 208 genera. All 14 families have larvae with venomous hairs (setae) and/or spines; yet only 4 families (Erebidae, Notodontidae, Saturniidae, and Zygaenidae) include venomous adult moths, which occur exclusively in females (Battisti et al. 2024). The venomous nature of both larvae and adults is thought to be a defense against natural enemies (Battisti et al. 2024).

Adverse reactions caused by direct or indirect contact with lepidopteran larvae or pupae are known as erucism, and with adults as lepidopterism (Pesce and Delgado 1971, Kawamoto and Kumada 1984). Battisti et al. (2011) defined 3 types of urticating structures in arthropods: true setae (detachable), modified setae (non-detachable), and spines (non-detachable). Both erucism and lepidopterism can be caused by contact with urticating true setae that are dislodged and airborne (Mullen and Zaspel 2019). True setae, along with modified setae and spines, can be transferred from larvae to cocoons, pupae, and adults (Mullen and Zaspel 2019), highlighting the multifunctional defensive role of these structures (Battisti et al. 2024). In adult females, urticating true setae occurring alone or in combination with non-urticating scales are found on the seventh abdominal tergite, called the corethrogyne or anal tuft (Davis et al. 2008, Battisti et al. 2017). In *Euproctis* spp. (Erebidae), adult females may acquire true setae shed from the larva as it emerges from the cocoon and combine them with other setae produced in the corethrogyne (Kemper 1955, Tsutsumi 1958, Wirtz 1984, Ellis et al. 2021). Urticating true setae produced by adult females tend to be longer, thinner, and more curved than those of larvae (Lamy 1984, Battisti et al. 2017). The corethrogyne setae are used to cover the egg mass, protecting the eggs and the young larval colony (Davis et al. 2008, Battisti et al. 2017).

Despite the ecological and medical significance of adult urticating setae, most studies have concentrated on the larval stage, which is more frequently encountered by humans and domestic animals. Perhaps the best-known case of urticating moths is the genus *Hylesia* (Saturniidae), with outbreaks occurring in South America and the Caribbean, where the moths are attracted to the lights and cause dermatitis in humans (Mullen and Zaspel 2019, Casafús et al. 2022). Other evidence of severe impact on human health is provided for *Gazalina* Walker in Nepal, where a destructive intraocular inflammatory disease, Seasonal Hyperacute Panuveitis (SHAPU), was described (Upadhyay and Shrestha 2017). Based on currently available literature, the earliest morphological description with diagrams of urticating moth setae in Notodontidae appears to be that of *Anaphe* spp. Walker reported by Pomeroy (1921), followed by the first scanning electron microscopy (SEM) study of moth setae from *Anaphe venata* Butler by Rothschild et al. (1970). Hinton (1981) had opposing views to those of the aforementioned authors, proposing that the urticating setae of *Anaphe* and possibly *Epicoma* Hübner were derived from the larva and picked up by the moth. To date, there have been no further investigations of adult urticating true setae in multiple notodontid genera.

This study addresses this lacuna by comparing the morphological structures of urticating true setae in adult processionary moths of Notodontidae. The non-urticating scales present in the corethrogyne were excluded from this study, which focused only on the urticating true setae. Of the 31 genera previously classified within Thaumetopoeinae (Kobayashi and Nonaka 2016), now split into Anaphinae and Thaumetopoeinae following the reclassification by St Laurent et al. (2025), 8 have been recorded to include urticating adults. The study by St Laurent et al. (2025), which analyzed up to 854 anchored hybrid enrichment loci from 150 species, addressed taxonomic uncertainties within Notodontidae and established a revised subfamily-level classification. The present study applies that updated phylogeny to compare the morphology of 14 notodontid species from 10 genera across the 2 subfamilies. The 10 genera included in this study are 5 genera of Anaphinae (*Adrallia* Walker, *Anaphe*, *Epanaphe* Aurivillius, *Hypsoides* Butler, and *Paradrallia* Bethune-Baker) and 5 genera of Thaumetopoeinae (*Epicoma*, *Gazalina*, *Ochrogaster* Herrich-Schäffer, *Tanystola* Turner, and *Trichiocercus* Stephens). The genera examined were selected based on published records of lepidopterism and broad biogeographic distributions. While *Adrallia* and *Trichiocercus* have not previously been recorded to have urticating true setae as adults, their inclusion provides an opportunity to assess morphological variation or similarity within the family. Most literature on lepidopterism in Notodontidae is derived from medical and biological publications rather than taxonomic descriptions. This is the first study to systematically compare adult female urticating setae across Notodontidae, providing morphological insights that may inform both taxonomic distinctions and hypotheses on defensive function. Such studies lay the groundwork to better understand the evolutionary and functional significance of these structures, whether they serve to defend short-lived females, protect developing offspring, or both. The diversity in setal morphology provides some clues.

## Materials and Methods

The female corethrogyne material from all genera of Notodontidae except *Ochrogaster* was retrieved from the Lepidoptera collection of the British Natural History Museum (BNHM) in London, United Kingdom (Table 1; Supplementary Fig. S1). The species examined were *Adrallia bipunctata* Walker; *Anaphe panda* Boisduval; *Anaphe reticulata* Walker; *An. venata*  *Epanaphe moloneyi* Druce; *Epicoma argentata* Walker; *Epicoma signata* Walker; *Gazalina chrysolopha* Kollar; *Hypsoides bipars* Butler; *Hypsoides* cf. *meloui* Oberthür; *Paradrallia punctigera* Hulstaert; *Tanystola isabella* White; and *Trichiocercus sparshalli* (Curtis). *Ochrogaster lunifer* Herrich-Schäffer comprises multiple undescribed species (Mills 1952, Common 1990, Floater 1996, Floater and Zalucki 2000, Mather et al. 2019); therefore, specimens from The University of Queensland (UQ), identified by nesting form and host plant, were used (see Perkins et al. 2016). Most BNHM specimens were collected over 100 years ago and were fragile; therefore, it was not possible to loan a whole specimen for each species to do SEM and light microscopy (LM) imaging. To address this, a small clump of the corethrogyne setae was removed from each specimen and loaned from the museum to take SEM and LM images. How the setae are embedded in the female abdomen could not to be imaged in this study. A small clump of the corethrogyne from the female was collected by gently brushing a fine paint brush against the tuft in an upward rolling motion; the tuft material easily becomes dislodged from the abdomen. The tuft material of each specimen was put in a labeled 1.5 ml Eppendorf tube containing 70% ethanol to prevent the urticating setae from becoming airborne. The paint brush was cleaned with ethanol and checked under the stereomicroscope to ensure there was no cross-contamination between the specimens.

**Table 1.** Female Notodontidae (Anaphinae and Thaumetopoeinae) corethrogyne true setae characteristics ^a^

Subfamily	Species	Proximal tip	Distal end	Length (mean ± SE)	Range	Description	General appearance
**Anaphinae**	*Adrallia bipunctata* 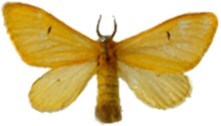	Lobed	Tapered + verrucose Spatulate	1.13 ± 0.05 mm (*N* = 21) 0.92 ± 0.05 mm (*N* = 2)	0.63 to 1.46 mm 0.87 to 0.97 mm	Two types of setae: (i) Seta of variable lengths with a ∼160° bend at the midpoint. Proximal tip bears widely spaced rows of lobed barbs that become sharper and resembles barbed wire along the first two thirds of the seta. Distal one-third has fewer barbs and the seta narrows and twists, terminating in a tapered distal end with verrucose projections. (ii) Seta with numerous overlapping lobed barbs from the proximal tip to one-eighth of the seta. The seta flattens and the lobed barbs transition into raised parallel rows of overlapping elongated barbs. Between these rows, regularly spaced transverse ridges are present. At the setal midpoint, the outermost rows of elongated barbs continue along the length while the inner rows of barbs flatten. The parallel rows of barbs smoothen, and forms raised ridges as the seta terminates in a spatulate structure.	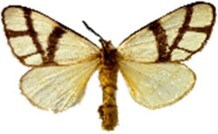 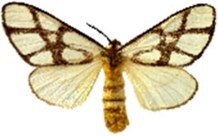
	*Anaphe panda* 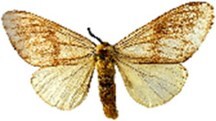	Serrate	Tapered + verrucose	1.06 ± 0.04 mm (*N* = 30)	0.64 to 2.21 mm	Single type of seta of variable lengths with ∼160° bend at two-fifths of its length. The proximal tip bears continuous serrated barbs in a single file, which subsequently forms 3 ridges. In the distal one-eighth of the seta, the barbs smoothen, and the seta tapers to a rounded apex bearing parallel ridges and verrucose projections.	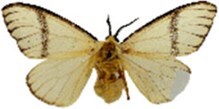
	*Anaphe reticulata* 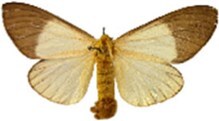	Serrate	Tapered + verrucose	1.66 ± 0.07 mm (*N* = 30)	1.11 to 2.42 mm	Same as *An. panda* but with ∼144° bend at the midpoint. The distal one-fourth of the seta tapers into a thin rod and terminates as a rounded apex bearing parallel ridges and verrucose projections.	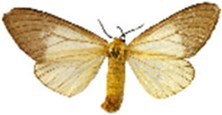
	*Anaphe venata* 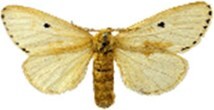	Serrate	Tapered + verrucose	1.74 ± 0.04 mm (*N* = 26)	0.96 to 1.96 mm	Same as *An. panda* but the ∼160° bend occurs at the midpoint, and the serrated barbs smoothen over the distal three-fourth of the seta.	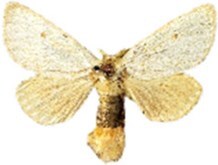
	*Epanaphe moloneyi* 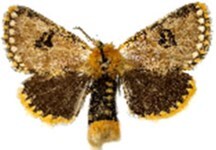	Serrate	Spatulate + verrucose	1.07 ± 0.09 mm (*N* = 22)	0.59 to 1.82 mm	Single type of seta of variable lengths with ∼156° bend at the midpoint. The proximal tip bears rows of serrated barbs, each of which transitions into raised parallel rows of overlapping elongated barbs. Between these parallel rows, numerous and regularly spaced transverse ridges are present. The parallel rows and transverse ridges gradually smooth and terminate in a spatulate structure bearing verrucose projections.	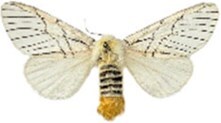
	*Hypsoides bipars* 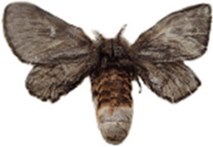	Serrate	Tapered + verrucose Spatulate + verrucose	0.65 mm (*N* = 1) 1.56 ± 0.05 mm (*N* = 14)	N/A 1.27 to 1.87 mm	Two types of setae: (i) Short, relatively straight seta with a tapered distal end. The proximal tip bears serrated barbs arranged in 3 rows, which form 3 well-defined barbed ridges along most of the seta’s length. The surface between these ridges remains smooth. In the distal one-fourth of the seta, the barbs gradually smooth, the seta narrows and twists and terminates in a tapered, rounded apex with distinct ridges and numerous verrucose projections. (ii) Long seta structurally similar to the short seta but with a ∼140° bend at the midpoint and a spatulate distal apex. The distal three-fourth of the seta tapers into a thin rod before terminating in a spatulate structure bearing defined ridges and numerous verrucose projections.	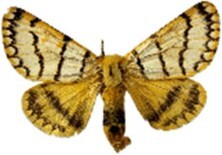 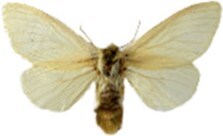
	*Hypsoides* cf. *meloui* 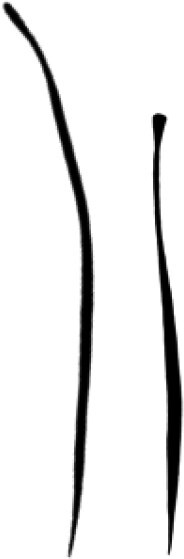	Serrate	Tapered + verrucose Spatulate + verrucose	0.85 ± 0.07 mm (*N* = 21) 1.75 ± 0.05 mm (*N* = 12)	0.33 to 1.33 mm 1.48 to 1.94 mm	Same as *H. bipars* but the spatulate distal structure has 2 short and straight projections at the apex.	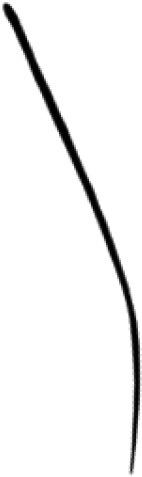
	*Paradrallia punctigera* 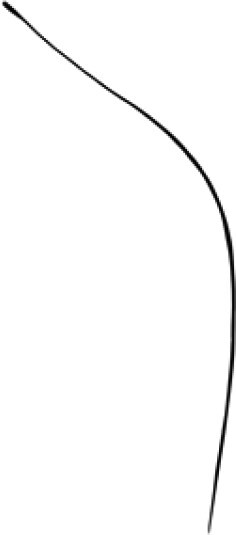	Lobed	Tapered Blunt	0.66 ± 0.01 mm (*N* = 16) 0.40 ± 0.01 mm (*N* = 9)	0.58 to 0.74 mm 0.35 to 0.48 mm	Two types of setae: (i) Symmetrical seta with a ∼160° bend at the midpoint. The proximal tip bears overlapping lobed barbs, which transition into rows of barbs continuing along the length of the seta, forming multiple single-file ridges. Between these ridges, a single line of irregularly sized small holes runs longitudinally. This structure persists throughout the entire length of the seta until it tapers and terminates in a fine apex. (ii) Short, wide, and straight seta with lobed barbs extending from the proximal tip along the entire length of the seta. The spacing between the lobed barbs increases with the widening of the seta. In the distal one-sixteenth of the seta, 2 longitudinal lines of irregularly sized small holes are present between the lobed barbs, and the seta terminates in a blunt apex.	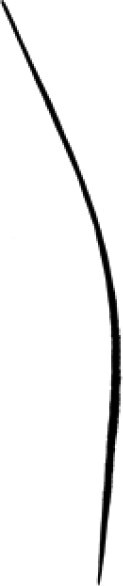
**Thaumetopoeinae**	*Epicoma argentata* 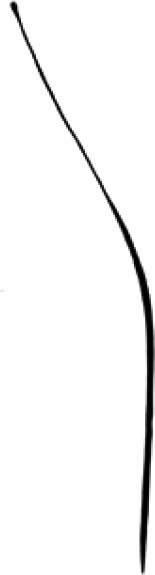	Lobed	Spatulate + verrucose	1.29 ± 0.09 mm (*N* = 14)	0.83 to 2.28 mm	Single type of seta of variable lengths with a ∼160° bend at the midpoint. The proximal tip bears overlapping lobed barbs for one-sixteenth of its length. The seta then flattens, with the inner barbs becoming smooth and losing their barbed structure, while the outer barbs transition into ridges composed of parallel rows of overlapping barbs. This arrangement continues toward the distal end, which terminates in a slightly spatulate structure, characterized by rows of raised ridges separated by single rows of irregularly sized small holes and verrucose projections.	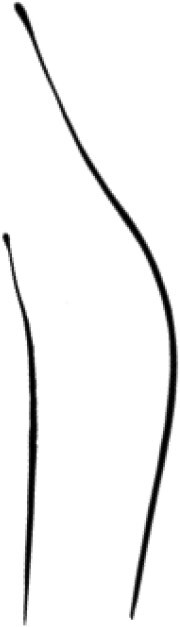
	*Epicoma signata* 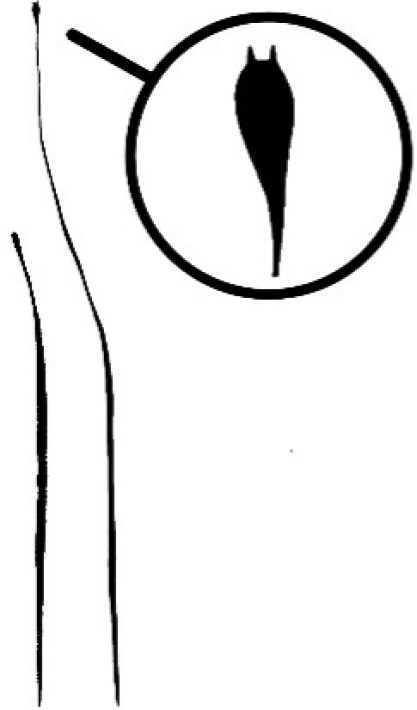	Lobed	Spatulate + verrucose	1.39 ± 0.09 mm (*N* = 13)	0.87 to 1.80 mm	Same as *Epi. argentata* but the bend at the midpoint is ∼152°.	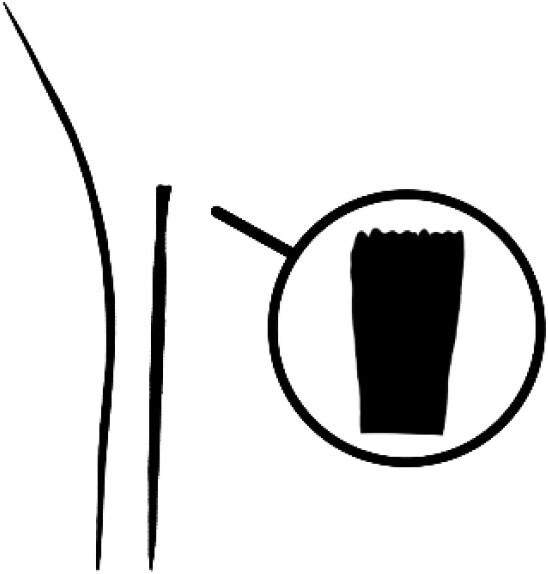
	*Gazalina chrysolopha* 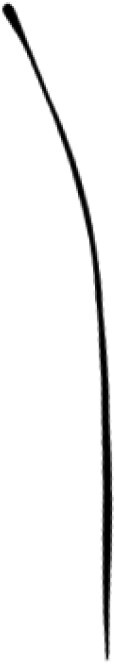	Lobed	Hook	0.77 ± 0.02 mm (*N* = 9)	0.70 to 0.86 mm	Single type of straight seta with a hook-like tapered distal end. Proximal tip bears overlapping lobed barbs that elongate and continue along the length of the seta. In the distal one-eighth, the seta tapers and abruptly bends into a hook.	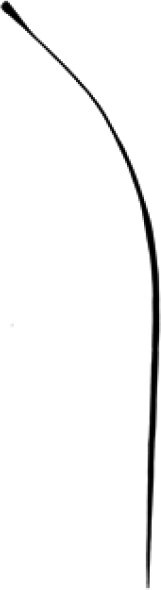
	*Ochrogaster lunifer* 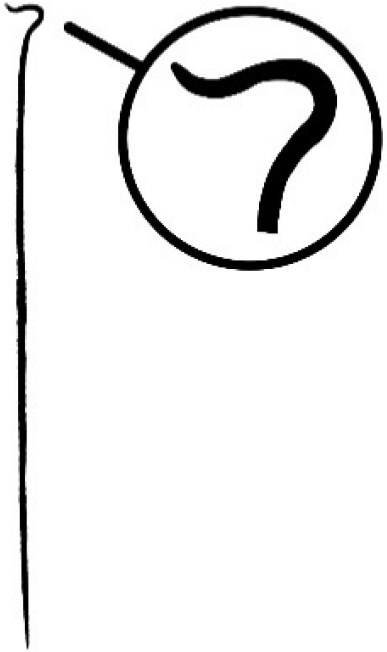	Lobed	Cup	1.98 ± 0.02 mm (*N* = 9)	1.84 to 2.09 mm	Single type of long filamentous seta with a cup-like structure at the distal end. The proximal tip bears overlapping lobed barbs. After the first one-eighth of the length, the lobed barbs become elongate with sharper distal ends that fan outward, and this arrangement continues for three-fourth of the setal length. In the distal one-eighth of the seta, both the barbs and setal width narrows, eventually flattening into a scale-like structure. The scale-like structure exhibits irregular undulations and fine striations, producing a wrinkled surface texture. The seta terminates abruptly in a cup-like structure.	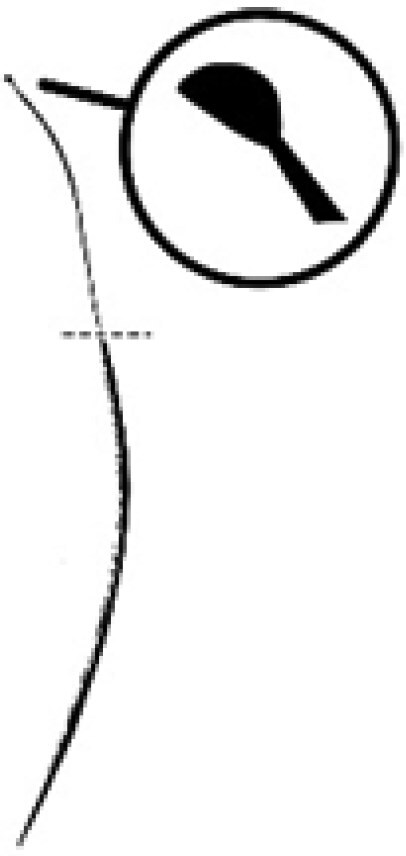
	*Tanystola isabella*	Lobed	Tapered	3.41 ± 0.08 mm (*N* = 8)	3.21 to 3.96 mm	Single type of long seta with a ∼150° bend at the midpoint. The proximal tip bears overlapping lobed barbs which transition into 3 well-defined ridges, forming a triangular transverse profile, while the other rows of lobed barbs between the ridges smoothen. This morphology continues for two-thirds of the length after which it narrows into a fine filament and terminates in a tapered apex.	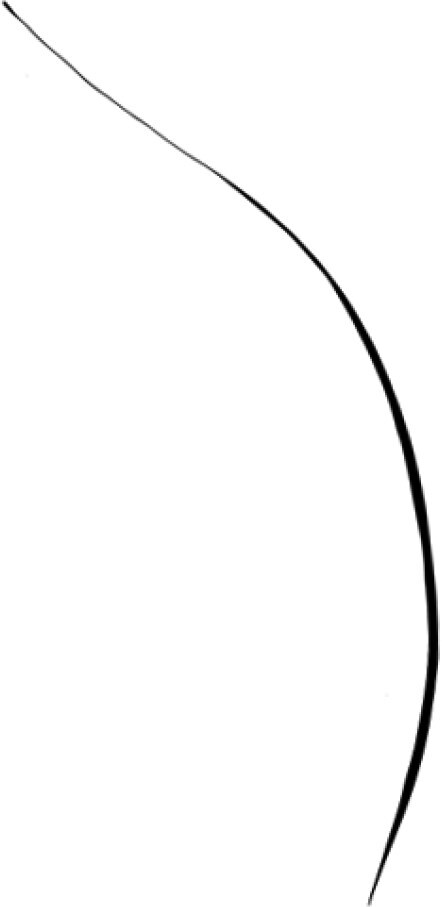
	*Trichiocercus sparshalli*	Lobed	Spatulate	1.19 ± 0.04 mm (*N* = 34)	0.69 to 1.59 mm	Single type of thick, flat seta with a ∼170° bend at three-fourth of its length. The proximal tip bears overlapping lobed barbs which transition into numerous rows of overlapping barbs at one-eighth of the length as the seta flattens. This structural arrangement continues along the length of the seta. Toward the distal end, the seta gradually narrows before broadening into a spatulate structure, where rows of barbs transform into well-defined smooth ridges. Between these ridges, numerous fine transverse lines run along the ridges and terminates in a spatulate structure. This setal morphology is very similar to *Ad. bipunctata*.	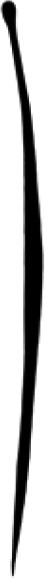

a Images and diagrams not to scale. All images are of actual specimens; some discoloration may have resulted from prolonged curation. Setae diagrams were drawn from light micrographs of the same specimens.

### Scanning Electron Microscopy

Some of the corethrogyne material was removed from the Eppendorf tube using featherweight forceps and placed on a filter paper inside a 4 cm diameter plastic Petri dish enclosed with a lid. Drying the corethrogyne material from the ethanol within the Petri dish minimized the risk of urticating true setae becoming airborne. After approximately 10 min, the dried material was transferred onto an SEM stub with a carbon tab using featherweight forceps. The scales and setae were gently flattened on the stub to prevent dislodgement within the vacuum chamber. Each SEM stub sample was cleaned with ultraviolet light and then sputter-coated with 15 nm of platinum using the platinum coater Q150TS. The SEM images were taken by Centre for Microscopy and Microanalysis staff at UQ using the Field Emission Scanning Electron Microscope JEOL 7100F. Additional SEM images were taken using a Phenom Desktop SEM.

### Light Microscopy

The same drying procedure described above was followed to prepare slide mounts of the corethrogyne material. A few scales and setae were transferred onto a slide using featherweight forceps and mounted in Hoyer’s, covered with a 13 mm diameter coverslip. The slides were kept in an oven at 40 °C for 2 wk. The slides were viewed under the Zeiss Axioskop 2 FS (Carl Zeiss, Jena, Germany) compound light microscope, and the LM images were captured and analyzed using the ToupView software (version 4.12.27501, ToupTek China).

### Analyses

Morphological characteristics of the true setae from all notodontid female corethrogyne were examined using descriptive analyses based on SEM and LM images. Lengths of intact true setae were measured from LM images to the nearest 0.00001 mm using the segmented line tool in ImageJ software (version 1.53 m, National Institutes of Health, United States). The mean and standard error of the setal length were calculated for each species and reported to the nearest 0.01 mm. To provide a molecular framework for comparing setal morphologies across taxa, a phylogenetic tree was constructed using publicly available cytochrome c oxidase subunit I (COI) gene sequences from the Barcode of Life Data Systems (BOLD). The COI region was selected as it is a widely sequenced mitochondrial marker for Lepidoptera; however, sequences were not available for all species of Notodontidae included in this study. Sequences were retrieved for all species except *Ad. bipunctata* (no suitable substitute), *P. punctigera* (substituted with *P. rhodesi*), *H. bipars* and *H.* cf. *meloui* (substituted with *Hypsoides* sp., *Hypsoides antsianakana* and *Hypsoides conglomerata*). *Thaumetopoea*, one of the most widely known thaumetopoeine genera, was included in the tree as a non-urticating moth outgroup. When available, sequences belonging to species within the subfamilies Anaphinae and Thaumetopoeinae were selected from at least 2 different specimens per species, preferably representing different Barcode Index Numbers and prioritizing sequences longer than 500 bp (corresponding to the standard barcode region of the COI gene). A full list of reference sequences used for phylogenetic tree construction is provided in Supplementary Table S1. Phylogenetic analyses were conducted using the Maximum Likelihood approach implemented in IQ-TREE v3.0.1 (Wong et al. 2026), following the protocol described in Basso et al. (2017, 2023). ModelFinder was employed to determine the best-fitting nucleotide substitution model based on the Bayesian Information Criterion (BIC) (Kalyaanamoorthy et al. 2017). A total of 104 sequences were aligned using MAFFT, as implemented in the TranslatorX pipeline (Abascal et al. 2010, http://www.translatorx.co.uk/). Longer sequences were trimmed to the 5′ barcode region of the COI mitochondrial gene, resulting in a final alignment spanning 658 base pairs. Twenty independent runs were performed using the parameters described above, and log-likelihood scores were compared to identify the best-scoring tree topology. Node support was assessed using Ultrafast Bootstrap (UFB) (Minh et al. 2013, Hoang et al. 2018), while branch support was evaluated using the SH-like approximate likelihood ratio test (SH-aLRT) (Guindon et al. 2010). Values ≥90 were considered well supported. The phylogenetic analysis of the mitochondrial DNA barcode region was performed using the TIM2 + F + R4 nucleotide substitution model selected according to the BIC. The tree was rooted on the *O. lunifer* clade and was selected as a consistent reference point within Notodontidae, based on its placement in previous molecular and phylogenomic analyses (Regier et al. 2016, St Laurent et al. 2025).

## Results

**Fig. 1.**
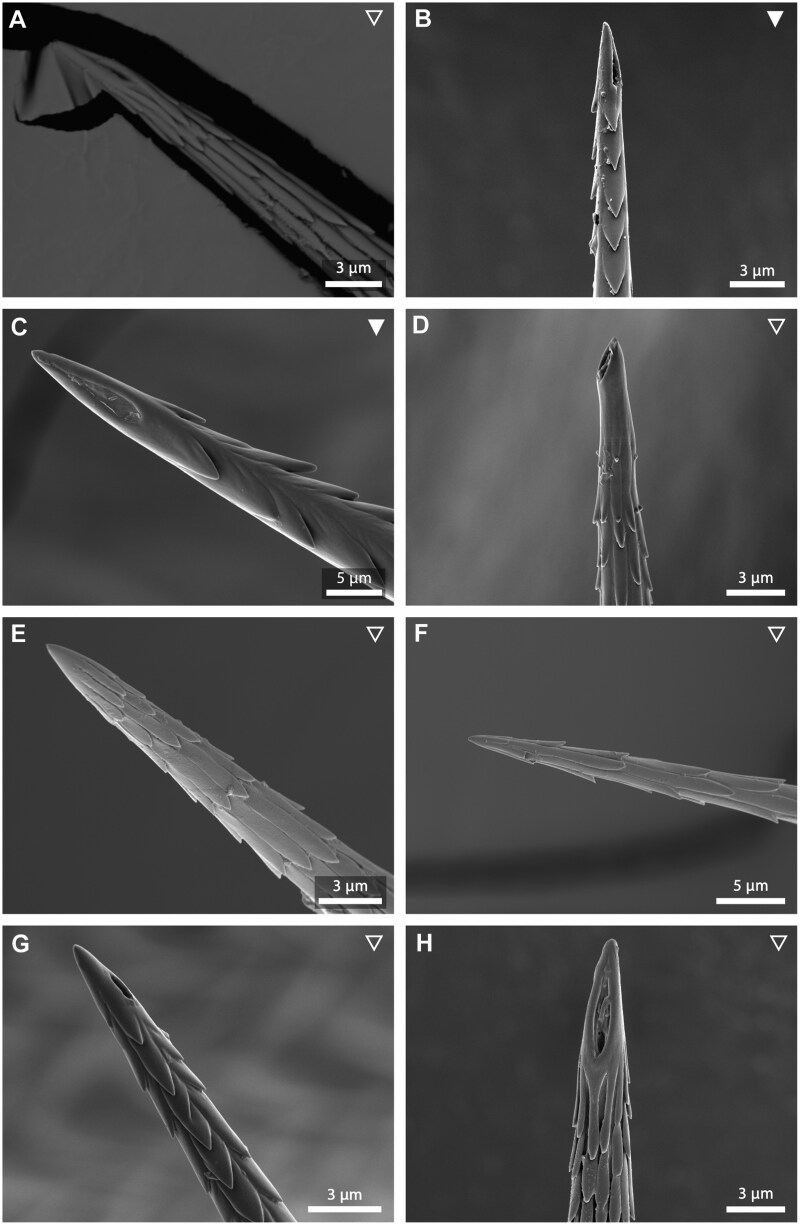
Scanning electron microscopy (SEM) images of the proximal tip of true setae from the female corethrogyne of 8 species of Notodontidae. Proximal tip morphology showing either lobed (▽) or serrated (▼) barbs. Anaphinae: A) *Adrallia bipunctata*, B) *Anaphe panda*, C) *Hypsoides* cf. *meloui*, and D) *Paradrallia punctigera*. Thaumetopoeinae: E) *Epicoma signata*, F) *Gazalina chrysolopha*, G) *Ochrogaster lunifer*, and H) *Tanystola isabella*.

**Fig. 2.**
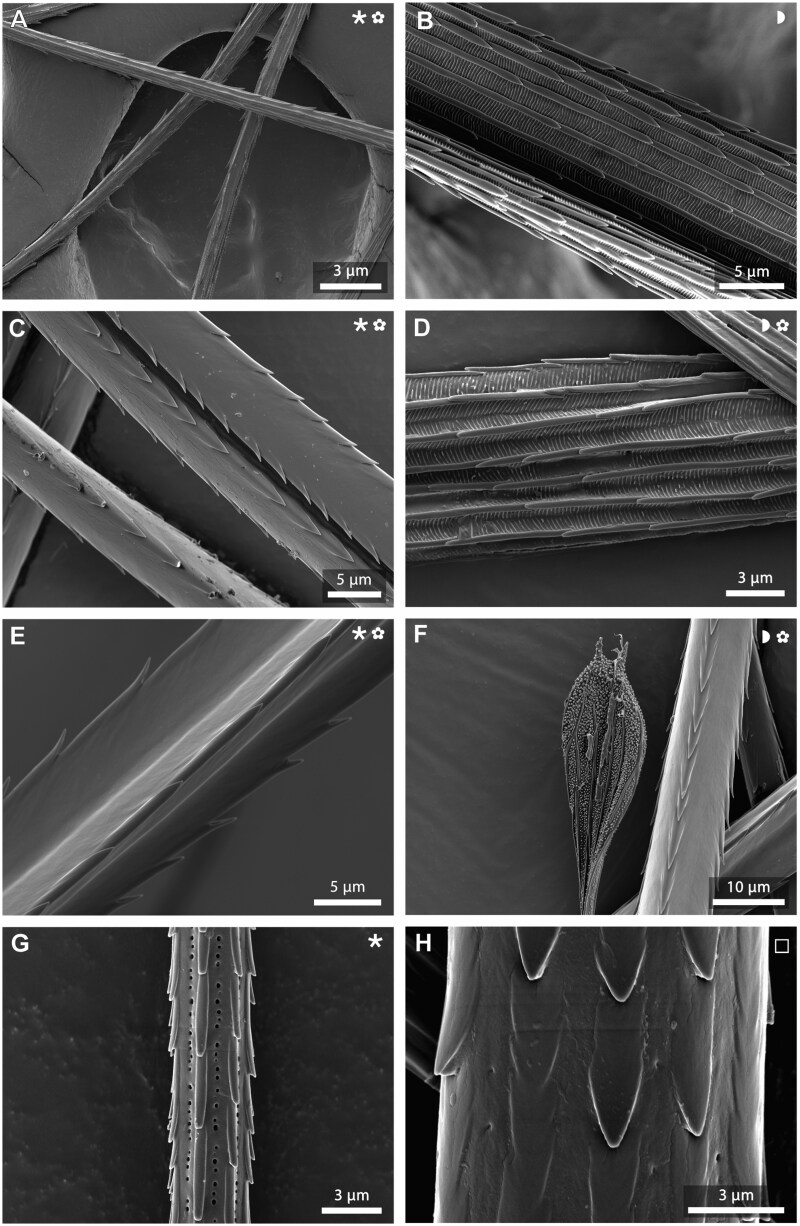
Scanning electron microscopy (SEM) images of the midpoint of true setae from 5 species of Anaphinae. Midpoint morphologies of tapered (*), spatulate (◗), blunt (◻), and verrucose (✿) setae. A and B) *Adrallia bipunctata*, C) *Anaphe panda*, D) *Epanaphe moloneyi*, E) *Hypsoides bipars*, F) *H*. cf. *meloui*, and G and H) *Paradrallia punctigera*.

**Fig. 3.**
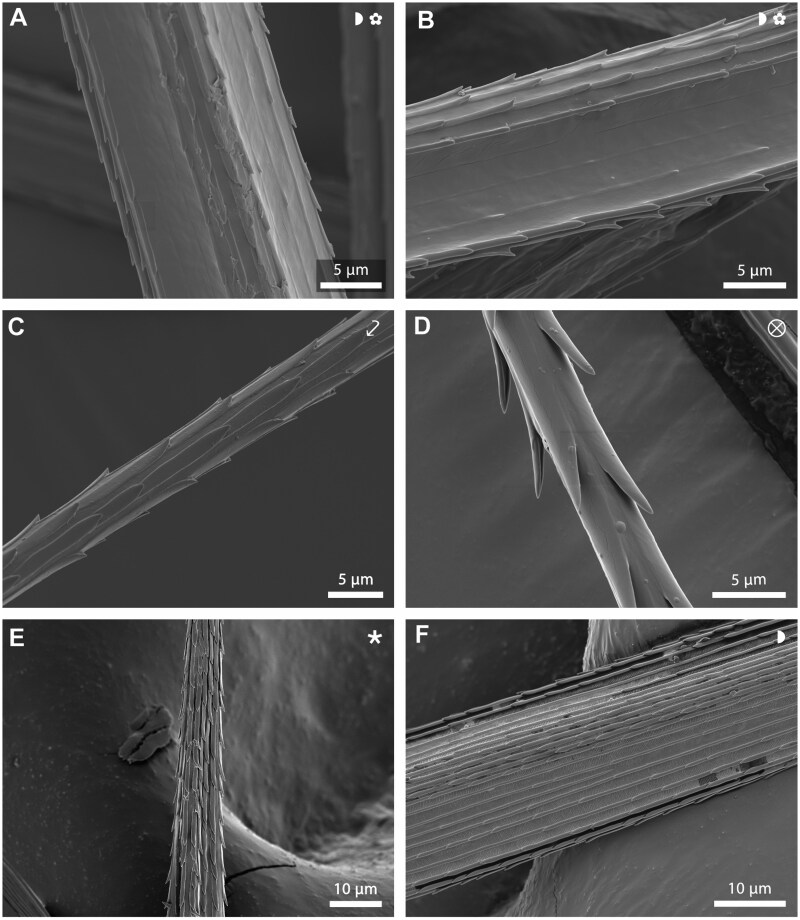
Scanning electron microscopy (SEM) images of the midpoint of true setae from 6 species of Thaumetopoeinae. Midpoint morphologies of spatulate (◗), hook-shaped (⤦), cup-shaped (Υ), tapered (*), and verrucose (✿) setae. A) *Epicoma argentata*, B) *Epi. signata*, C) *Gazalina chrysolopha*, D) *Ochrogaster lunifer*, E) *Tanystola isabella*, and F) *Trichiocercus sparshalli*.

**Fig. 4.**
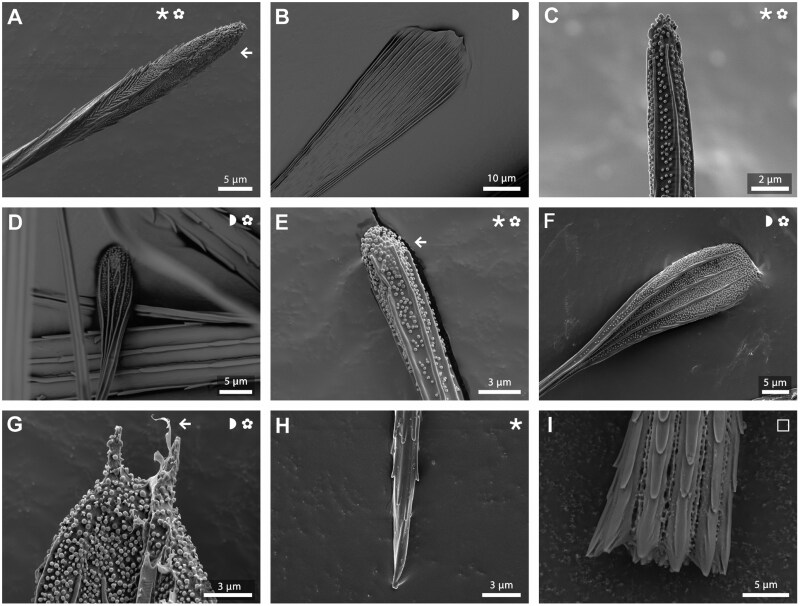
Scanning electron microscopy (SEM) images of the distal end of the true setae from 6 species of Anaphinae. Distal end morphologies include tapered (*), spatulate (◗), blunt (◻), and verrucose (✿) setae; with secretion (**←**). A and B) *Adrallia bipunctata*, C) *Anaphe panda*, D) *Epanaphe moloneyi*, E and F) *Hypsoides bipars*, G) *H*. cf. *meloui*, and H and I) *Paradrallia punctigera*.

**Fig. 5.**
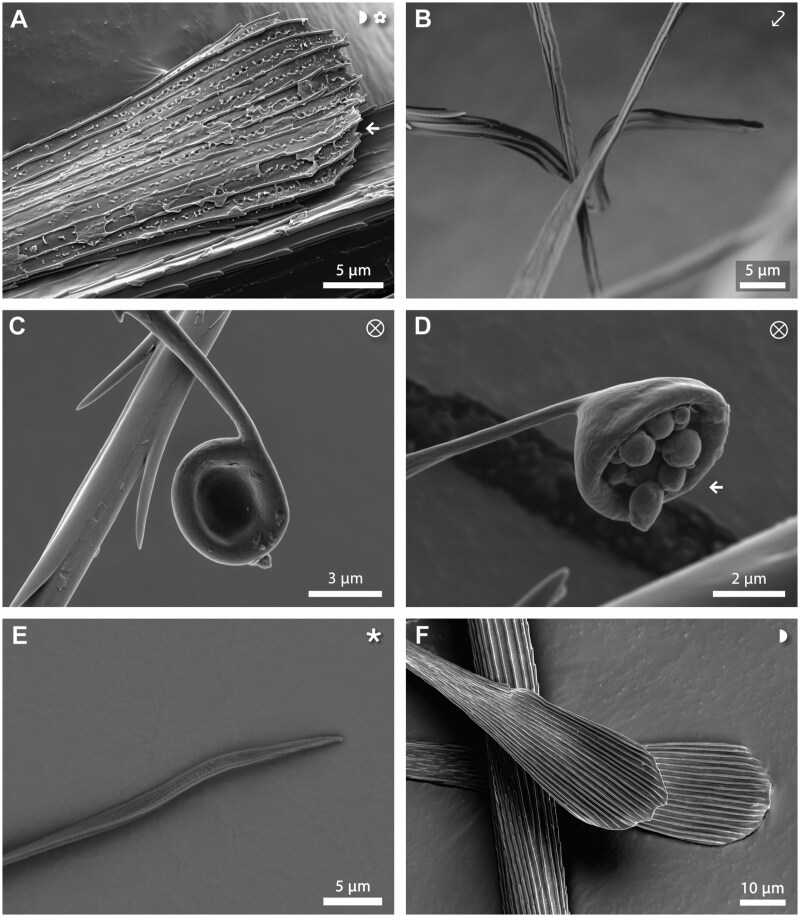
Scanning electron microscopy (SEM) images of the distal end of the true setae from 5 species of Thaumetopoeinae. Distal end morphologies include spatulate (◗), hook-shaped (⤦), cup-shaped (Υ), tapered (*), and verrucose (✿) setae; with secretion (**←**). A) *Epicoma argentata*, B) *Gazalina chrysolopha*, C and D) *Ochrogaster lunifer*, E) *Tanystola isabella*, and F) *Trichiocercus sparshalli*.

The corethrogyne material from all 14 species of Notodontidae examined had various types of true setae (Figs. 1 to 5; for all species comparison, see Supplementary Fig. S2). In all species, the proximal tip of the true seta is spear-like, with a lateral opening (Fig. 1). The proximal tip was followed by rows of serrated or lobed barbs, which directed toward the distal end. Serrated barbs were wide and triangular in shape, whereas lobed barbs were elongated and distally more rounded. The variation of setal morphology was most evident in the distal end of the seta, which fell into 5 morphological categories: tapered, spatulate, blunt, cup-shaped, or hook-shaped (Figs. 4 and 5). All species of Anaphinae except *P. punctigera* had dense verrucose nanostructures in the distal end of the seta (Fig. 4A, C to G). Whereas in Thaumetopoeinae, only *Epicoma* spp. had minimal verrucose nanostructures (Fig. 5A). Secretion-like substances covered the distal setal region in *Ad. bipunctata*, *H. bipars*, *H.* cf. *meloui*, *Epi*. *argentata*, and *O. lunifer* (indicated by **←** in Figs. 4A, E, and G and 5A and D, respectively). Irregularly sized small holes were present on the distal end of *P. punctigera*, *Epi. argentata*, and *Epi. signata*. Setae from all species observed were transparent and hollow along their entire length when viewed under LM. The length and overall setal morphology for each species are described and summarized in Tables 1 and 2. Descriptions of the 5 distal end characteristics are detailed below.

**Table 2.** Summarized table of Notodontidae female moth true setae

Subfamily	Genera	Proximal tip	Distal structure				
			Tapered	Spatulate	Blunt	Cup	Hook
**Anaphinae**	*Adrallia*	Lobed	Π	Π			
	*Anaphe*	Serrate	Π				
	*Epanaphe*	Serrate		Π			
	*Hypsoides*	Serrate	Π	Π			
	*Paradrallia*	Lobed	Π		Π		
**Thaumetopoeinae**	*Epicoma*	Lobed		Π			
	*Gazalina*	Lobed					Π
	*Ochrogaster*	Lobed				Π	
	*Tanystola*	Lobed	Π				
	*Trichiocercus*	Lobed		Π			

The tick represents the setal characteristic that is present, and the gray shading represents the presence of verrucose projections.

### Tapered

The most common form. The distal end of the seta gradually narrows to a rounded or finer tip with multiple parallel longitudinal ridges. The verrucose forms have numerous circular projections between the ridges. Tapered setae are present in Anaphinae (*Ad. bipunctata*, *An. panda*, *An. reticulata*, *An. venata*, *H. bipars, H.* cf. *meloui*, and *P. punctigera*) (Fig. 4A, C, E, and H; Supplementary Fig. S2A to D and F to H) and Thaumetopoeinae (*Ta. isabella*) (Fig. 5E; Supplementary Fig. S2M).

### Spatulate

The second most common form. The distal end of the seta gradually widens into a broad, paddle-shaped structure with multiple parallel longitudinal ridges. The verrucose forms have numerous circular projections between the ridges. Spatulate setae are present in Anaphinae (*Ad. bipunctata*, *Epa. moloneyi*, *H. bipars*, and *H.* cf*. meloui*) (Fig. 4B, D, F, and G, respectively; Supplementary Fig. S2A and E to G) and Thaumetopoeinae (*Epi. argentata*, *Epi. signata*, and *Tr. sparshalli*) (Fig. 5A and F; Supplementary Fig. S2I, J, and N).

### Blunt

The distal end of the seta terminates as a blunt structure. This form is unique to *P. punctigera* (Fig. 4I; Supplementary Fig. S2H).

### Cup

The distal end of the seta terminates in a cup-like structure. This form is unique to *O. lunifer* (Fig. 5C and D; Supplementary Fig. S2L).

### Hook

The distal end of the seta gradually narrows and terminates as a hook shape. This form is unique to *G. chrysolopha* (Fig. 5B; Supplementary Fig. S2K).

**Fig. 6.**
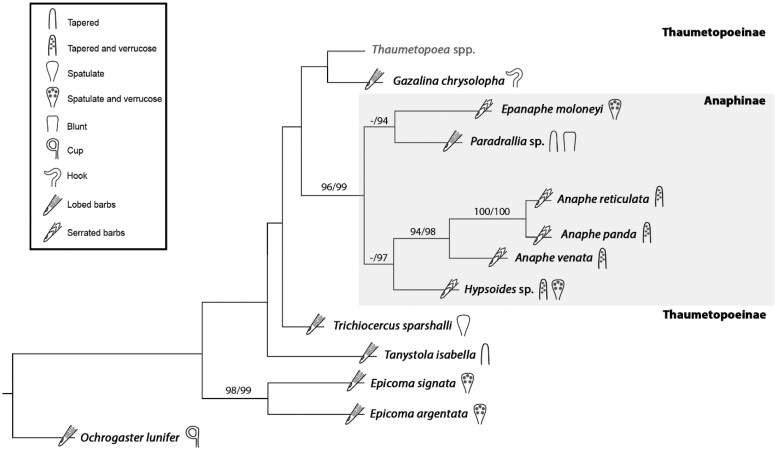
Maximum likelihood tree redrawn from Supplementary Fig. S3 of notodontid species examined in this study (excluding *Adrallia bipunctata*), with the inclusion of a non-urticating moth outgroup *Thaumetopoea* spp. (gray text). COI gene sequences were analyzed and only support values above 90% are shown at the nodes, representing SH-aLRT support (%)/ultrafast bootstrap support (%). Taxa within the shaded gray rectangle belong to the subfamily Anaphinae and taxa outside belong to Thaumetopoeinae. Each taxon is represented by icons illustrating the setal characteristics of the proximal tip and distal end. *Thaumetopoea* spp. does not have an icon of setal characteristics because they are non-urticating as adults.

The phylogenetic relationships among notodontid species were determined using COI sequences, and setal morphologies were illustrated onto the phylogenetic tree to examine patterns of morphological and geographic variation (Fig. 6; full tree with SH-aLRT and UFB support values in Supplementary Fig. S3). Nodes and branches along the backbone were generally poorly supported, whereas distal nodes, particularly those defining individual genera, showed high support values (>90%) and were considered well supported. Although BOLD COI sequences were not available for all species in the study, 4 geographic clades were apparent: Afrotropical (*Anaphe*, *Epanaphe*, *Hypsoides*, and *Paradrallia*), Australasian (*Epicoma*, *Ochrogaster*, *Tanystola*, and *Trichiocercus*), Palearctic/Asian (*Gazalina*), and the Palearctic/Afrotropical (*Thaumetopoea*). The Afrotropical clade was strongly supported at terminal nodes (≥94%), and all genera were monophyletic. Subclades within these genera generally exhibited short branch lengths, indicating low intraspecific divergence. Morphologically, most genera in this clade shared a serrated proximal tip and verrucose distal end of the setae, except for *Paradrallia*, which lacked this pattern. In the Australasian clade, all genera were retrieved as monophyletic in this dataset. All genera in the Australasian clade shared a lobed proximal tip but differed in the distal end. The Palearctic/Asian clade, represented by *Gazalina*, formed a distinct and strongly supported lineage (≥98%) with hook-shaped setae, clearly separated from the Afrotropical and Australasian clades. The Palearctic/Afrotropical clade, corresponding to *Thaumetopoea*, was largely monophyletic, with strong support for terminal nodes (>98%). Together with *Gazalina*, *Thaumetopoea* clustered near the Afrotropical clade, suggesting a closer evolutionary relationship within these lineages than with others. Overall, the tree reflects both geographic structuring and genetic divergence among genera.

## Discussion

Adult females of Anaphinae and Thaumetopoeinae (Notodontidae) are non-feeding and short-lived, typically surviving 2 and 3 d and usually lay a single egg mass. The urticating true setae on adult females may serve multiple functions: defense of the adults, dispersing setae into the environment to create a gradient of irritation (“sanitising” the area), and, more directly, protect the egg mass that has been laid. The morphological characteristics of the setae suggest all 3 may be possible.

This comparative morphological study of urticating true setae from the corethrogyne of 14 species of notodontid females revealed structural differences at the genus level. The setae from all genera had a spear-like proximal tip with a lateral opening. The opening is presumably how the toxins are drawn into the seta through capillary action (Rothschild et al. 1970). Kawamoto and Kumada (1984) described the mechanism in *Euproctis subflava* (Erebidae) larvae whereby electron-dense materials (toxins) produced by trichogen cells fill the setal cavity via continuous passage from the socket wall hole to the setal hole. The morphology and mechanism of how the setae are attached to the socket on the last abdominal segment were not determined in this study; see Kawamoto and Kumada (1984), Lamy (1984), and Rothschild et al. (1970) for descriptions. LM showed that all the urticating setae were hollow throughout the length of the seta, suggesting this is where the toxins are stored after detachment from the female moth. Although the setal toxins were not explored, morphological and structural differences from SEM and LM images provided insight into how the seta could assist with urtication and its ecological role in the environment. Morphological differences were most evident in the distal end of the seta and were categorized into 5 forms: tapered, spatulate, blunt, cup-shaped, or hook-shaped, each with or without verrucose structures.

The tapered distal end was the most common morphological form of urticating setae, present in 8 of 14 species examined, followed by spatulate setae in 7 species. Three genera possessed 2 types of setae, tapered and spatulate in *Adrallia* and *Hypsoides* and tapered and blunt in *Paradrallia*. Despite the differences in distal structure, all setal types within each species shared the same proximal tip barb arrangement. All other genera had 1 type of distal morphology (tapered, spatulate, cup-shaped, or hook-shaped). Most genera had different lengths of setae even though they were structurally similar within the species. These distal structural types, coupled with variation in setal length, suggest potential ecological and functional differentiation. In studies of urticating larvae, variability in setae lengths resulted in dispersal differences, with smaller setae traveling up to 5 times further than larger setae (Petrucco Toffolo et al. 2014, Perkins et al. 2019). *Tanystola isabella* had the longest setal length among the species examined, averaging 3.41 mm. Rothschild (1970) described how the long thread-like setae in *Ta. isabella* (as *E. isabella*) entangle any object with which they come into contact. Rothschild (1970) mentioned the bulb-like expansion (spatulate) with infoldings (parallel longitudinal ridges) on the distal end of *Epa. moloneyi* setae has a sucker-like action, though the mechanism was not explained. The spatulate structure is most likely associated with facilitating lift and aerial dispersal. Across all species except *O. lunifer*, parallel longitudinal ridges extended through most of the setal length to the distal end, which may strengthen the seta whilst keeping it light weight and/or aid in adhering to eggs or surfaces of natural enemies. Personal observations during specimen handling confirmed that the corethrogyne material is easily airborne upon minimal disturbance. Among spiders of the subfamily Theraphosinae, light weight and aerodynamic urticating setae float through the air, targeting the respiratory tract of the attacker in the vicinity (Gawryszewski 2017), while shorter, thicker setae are only effective by direct contact (Perafán et al. 2016). In Lepidoptera larvae, urticating setae of *O. lunifer* (Perkins et al. 2019), Euproctis *chrysorrhoea* (Werno and Lamy 1994), and *Thaumetopoea* spp. (Werno and Lamy 1990, Fenk et al. 2007, Petrucco Toffolo et al. 2014) can travel several kilometers from the source, subject to environmental conditions. Variability in setal lengths may reflect functional specialization to different natural enemies, particularly vertebrates, and/or is related to different toxin types (Petrucco Toffolo et al. 2014).

Werno and Lamy (1994) determined an increase in urticating setae in the environment at times when female moths of *Eup. chrysorrhoea* were flying during reproductive activities, highlighting the effectiveness of passive release of urticating setae. A similar situation has been described for *Gazalina* in Nepal, where outbreaks of SHAPU, a destructive intraocular inflammatory disease, occur in humans when the moths are actively attracted to the lights (Upadhyay and Shrestha 2017). Rothschild et al. (1970) mentioned that the slightest contraction of a moth’s abdominal muscles can release the setae into the environment, potentially triggering adverse reactions in vertebrates. Recorded vertebrate predators for the species studied include bats and geckos for *Gazalina* (Pandey 2024), pied butcherbirds (*Cracticus nigrogularis*) for *Epicoma*, *Ochrogaster* (as *Teara contraria*), and *Trichiocercus* (Mackey 1985), raptors for *Epicoma* (Fuentes et al. 2024), and noisy miner birds (*Manorina melanocephala*) for *Ochrogaster* (pers. obs.). Airborne urticating setae may therefore deter some, but not all, aerial predators. Feeding experiments in Gabon showed that mandrills, egrets, and a mangabey rejected moths of *Anaphe* and *Epanaphe* as food due to repulsion (Bigot and Jouventin 1974). Notably, female moths were always rejected, while male moths were occasionally eaten, hypothesizing that males benefit from resembling females (Bigot and Jouventin 1974). This learned behavior indicates that predators associated wing pattern and aposematism with unpalatability (Bigot and Jouventin 1974). The brightly colored corethrogyne, together with wing patterns and/or aposematic coloration on the abdomen and/or wings in the species examined (Table 1; Supplementary Fig. S1; cf. non-aposematic moth of *Thaumetopoea* shown in Battisti et al. 2017), may serve as deterrent signals to diurnal predators such as birds and other visual predators. Additionally, the conspicuous golden corethrogyne present in most species likely enhances the visibility of the egg mass against the host plant bark to deter predators.

The phylogenetic tree constructed in this study provided a framework to compare setal morphology, genetic relatedness, and geographic variation in Notodontidae. The COI-based tree in this study was broadly consistent with the subfamily-level classification proposed by Regier et al. (2016) and St Laurent et al. (2025); however, relationships among some thaumetopoeine genera differed. This study was based on a single mitochondrial marker and should be interpreted with caution for determining deeper phylogenetic relationships. Nevertheless, the tree reflected geographic structuring, with Afrotropical, Australasian, Palearctic/African, and Palearctic/Asian lineages forming distinct clusters. The Afrotropical clade (*Anaphe*, *Epanaphe*, and *Hypsoides*) showed consistent traits, including serrated barbs proximally and verrucose distal ends, which may indicate a conserved adaptation. While *Paradrallia* is grouped within the Afrotropical clade, the seta displayed lobed barbs proximally and tapered or blunt distally with no verrucose structures, indicating a deviation in setal structure despite close genetic affinity. In contrast, the Australasian clade exhibited broader morphological diversity despite shared genetic ancestry, suggesting divergent ecological pressures linked to their biogeography. *Ochrogaster lunifer* was the most morphologically distinct with its cup-shaped setae, which were not observed in other taxa. The function of this cup structure is unknown and warrants further investigation. In 1 seta that was SEM imaged (Fig. 5D), secretion-like globules were observed within the cup, as discussed later. *Gazalina chrysolopha* was also unique morphologically with its hooked distal end, potentially an adaptation specific to the Palearctic/Asian lineage. The hook-shaped structure likely enhances mechanical entanglement, anchoring the seta onto potential natural enemies and eliciting an urticarial response. The placement of *Thaumetopoea* spp. near the Afrotropical taxa may reflect shared ancestry; however, their absence of urticating adult setae suggests a different egg defense strategy. Egg mass studies indicated that *Th. pityocampa* eggs have a thicker chorion compared to *O. lunifer*, which has a thinner chorion (pers. obs.). Investment in either a thicker chorion or urticating setae covering the egg mass may therefore represent alternative cost–benefit strategies shaped by ecological context.

The discovery of dense verrucose nanostructures covering the distal end of all anaphine species (*Ad. bipunctata*, *An. panda*, *An. reticulata*, *An. venata*, *Epa. moloneyi*, *H. bipars*, and *H.* cf. *meloui*) except *P. punctigera* suggests an adaptive trait with a functional role. In Thaumetopoeinae, verrucose distal end structures were observed in *Epi. argentata* and *Epi. signata*; however, the density of verrucose structures was much less. Lamy (1984) described “warts” at the distal end of *An. panda* and *An. venata* adult setae, but the reason for these structures was unclear. In Heteroptera, the scent gland systems have dense protuberant micro-sculptures that assist with the retention and slow dispersal of discharged secretion (Carver 1990, Durak and Kalender 2009). Most commonly, heteropterans, known as “stink bugs,” use secretion from their scent glands for defensive and pheromonal purposes (Durak and Kalender 2009). Although specialized scent gland systems comparable to those of Heteroptera have not been described in lepidopteran egg masses, these verrucose structures may potentially aid in the retention or gradual diffusion of defensive chemicals detectable to natural enemies. However, this remains unclear, and experimental work is required to fully understand the function of verrucose structures in Lepidoptera.

In at least 6 species (*Ad. bipunctata*, *An. reticulata*, *Epi*. *argentata*, *H. bipars*, *H.* cf. *meloui*, and *O. lunifer*), secretion-like substances covered the distal setal region. Rothschild et al. (1970) described the distal end of the seta from *Epanaphe*, *Anaphe*, *Gazalina*, and *Epicoma* as “modified to improve its adhesive qualities.” Irregularly sized small holes observed in the distal end of the seta from *P. punctigera* and *Epicoma* spp. and verrucose structures observed in 9 of the 14 notodontid species studied may indicate a secretory function. In *Hylesia metabus* (Lepidoptera: Saturniidae) females, the flattened scale-like urticating setae taper into a thin cylindrical tube from which secretion drops have been observed (Rodriguez et al. 2004). Biochemical studies of urticating setae from female corethrogyne of *Hyl. metabus* determined a kallikrein-like substance that causes urticaria (Lundberg et al. 2002). Lamy (1984) further noted that in *An. venata*, toxins could be released from the distal end without breaking the seta. As some of these specimens examined in this study were over 100 yr old, it is difficult to determine if these secretions hardened and remained intact this whole time. Further experimental work is required to determine the composition and function of secretions associated with true setae in the corethrogyne.

While not all urticating setal types may have been observed in some specimens due to the removal of corethrogyne material from a particular area, true setae were detected in all species examined. This suggests that urticating setae may be distributed throughout the corethrogyne. A uniform distribution could potentially ensure an even coverage of urticating setae on the egg mass, which may provide protection against natural enemies from multiple directions. Non-urticating scales likely provide a mechanical barrier or hide the eggs from natural enemies and environmental elements, while urticating true setae deter mainly vertebrates via chemical means (Floater and Zalucki 1999, Rodriguez et al. 2004). Despite the effortless collection of tuft material from the specimens, locating the intact distal end of the seta was challenging, likely due to degradation from specimen age and brittleness. The ease of breakage suggests this is functionally advantageous as it facilitates the release of toxins that were stored inside the seta (Southcott 1987). The spear-like proximal tip and the numerous barbs that continue along most of the setal length may induce immediate mechanical irritation, eliciting a scratching response in the recipient that facilitates deeper toxin penetration, resulting in urticaria (Lundberg et al. 2002).

This study provides the first comparative analyses of high-resolution SEM images, integrating morphological and phylogenetic data across multiple urticating notodontid moth species. Our results suggest that while certain traits are conserved within lineages, others have evolved independently, highlighting the complexity of genetic divergence, functional morphology, and ecological context in Notodontidae. The urticating setae of adult moths represent a distinctive adaptation among invertebrates, and many of the species examined here had not been morphologically described until now. Although adverse reactions to adult *Ad. bipunctata*, *Epi. argentata*, *Epi*. *signata*, *P. punctigera*, and *Tr. sparshalli* have not yet been reported, the morphological structures of their true setae observed in this study suggest that these species may cause urticarial effects in vertebrates. The distinct setal characteristics of each species suggest that the genus may be distinguished based on setal morphology. Furthermore, the ease with which urticating true setae are dislodged, their aerodynamic properties, shared structural features that may facilitate toxin secretion and/or dispersal, and variation in setal lengths together suggest that these moth species possess highly effective defensive adaptations. This provides urticating notodontids with both wide-scale environmental deterrence to discourage natural enemies from approaching and close-range protection of the moths and their egg mass.

## Supplementary Material

ieag051_Supplementary_Data

## References

[ieag051-B1] Abascal F , Zardoya R, Telford MJ. 2010. TranslatorX: multiple alignment of nucleotide sequences guided by amino acid translations. Nucleic Acids Res. 38:W7–W13. 10.1093/nar/gkq291 20435676 PMC2896173

[ieag051-B2] Basso A , Avtzis D, Burban C, et al 2023. The pine processionary moth *Thaumetopoea pityocampa* (Notodontidae) species complex: a phylogeny-based revision. ASP. 81:1031–1050. 10.3897/asp.81.e102928

[ieag051-B3] Basso A , Negrisolo E, Zilli A, et al 2017. A total evidence phylogeny for the processionary moths of the genus *Thaumetopoea* (Lepidoptera: Notodontidae: Thaumetopoeinae). Cladistics 33:557–573. 10.1111/cla.12181 34724760

[ieag051-B4] Battisti A , Holm G, Fagrell B, et al 2011. Urticating hairs in arthropods: their nature and medical significance. Annu. Rev. Entomol. 56:203–220. 10.1146/annurev-ento-120709-144844 20809805

[ieag051-B5] Battisti A , Larsson S, Roques A. 2017. Processionary moths and associated urtication risk: global change–driven effects. Annu. Rev. Entomol. 62:323–342. 10.1146/annurev-ento-031616-034918 27860523

[ieag051-B6] Battisti A , Walker A, Uemura M, et al 2024. Look but do not touch: the occurrence of venomous species across Lepidoptera. Entomologia 44:29–39. 10.1127/entomologia/2023/2295

[ieag051-B7] Bigot L , Jouventin P. 1974. Quelques experiences de comestibilite de Lepidopteres gabonais faites avec le mandrill, le cercocebe a joues grises et le garde-boeufs. Terre Vie 28:521–543.

[ieag051-B8] Carver M. 1990. Integumental morphology of the ventral thoracic scent gland system of *Poecilometis longicornis* (Dallas) (Hemiptera: Pentatomidae). Int J Insect Morphol Embryol 19:319–321. 10.1016/0020-7322(90)90017-J

[ieag051-B9] Casafús MG , Gritti MA, González KY, et al 2022. Unraveling the distinctive venomous features of the saturniid *Hylesia* sp.: an integrative approach of a public health concern in Argentina. Acta Trop. 231:106428. 10.1016/j.actatropica.2022.106428 35339435

[ieag051-B10] Common IFB. 1990. Host selection and oviposition. In: Moths of Australia. Melbourne University Press. p. 48.

[ieag051-B11] Davis DR , Quintero AD, Cambra TRA, et al 2008. Biology of a new Panamanian bagworm moth (Lepidoptera: Psychidae) with predatory larvae, and eggs individually wrapped in setal cases. Ann. Entomol. Soc. Am. 101:689–702. 10.1603/0013-8746(2008)101[689:BOANPB]2.0.CO;2

[ieag051-B12] Durak D , Kalender Y. 2009. Fine structure and chemical analysis of the metathoracic scent gland secretion in *Graphosoma lineatum* (Linnaeus, 1758) (Heteroptera, Pentatomidae). C. R. Biol. 332:34–42. 10.1016/j.crvi.2008.10.004 19200924

[ieag051-B13] Ellis CR , Elston DM, Hossler EW, et al 2021. What’s eating you? Caterpillars. Cutis 108:346–351. 10.12788/cutis.0406 35167790

[ieag051-B14] Fenk L , Vogel B, Horvath H. 2007. Dispersion of the bio-aerosol produced by the oak processionary moth. Aerobiologia (Bologna) 23:79–87. 10.1007/s10453-007-9053-3

[ieag051-B15] Floater GJ. 1996. Life history comparisons of ground- and canopy-nesting populations of *Ochrogaster lunifer* Herrich-Schäffer (Lepidoptera: Thaumetopoeidae): evidence for two species? Aust. J. Entomol. 35:223–230. 10.1111/j.1440-6055.1996.tb01395.x

[ieag051-B16] Floater GJ , Zalucki MP. 1999. Life tables of the processionary caterpillar *Ochrogaster lunifer* Herrich-Schäffer (Lepidoptera: Thaumetopoeidae) at local and regional scales. Aust. J. Entomol. 38:330–339. 10.1046/j.1440-6055.1999.00122.x

[ieag051-B17] Floater GJ , Zalucki MP. 2000. Habitat structure and egg distributions in the processionary caterpillar *Ochrogaster lunifer*: lessons for conservation and pest management. J. Appl. Ecol. 37:87–99. 10.1046/j.1365-2664.2000.00468.x

[ieag051-B18] Fuentes E , Olsen J, Debus S. 2024. Feeding ecology of the raptor guild of the Canberra region. Corella 48:1–23.

[ieag051-B19] Gawryszewski FM. 2017. Anti-predator strategies. In: Viera C, Gonzaga M, editors. Behaviour and ecology of spiders. Springer. p. 397–415.

[ieag051-B20] Guindon S , Dufayard JF, Lefort V, et al 2010. New algorithms and methods to estimate maximum-likelihood phylogenies: assessing the performance of PhyML 3.0. Syst. Biol. 59:307–321. 10.1093/sysbio/syq010 20525638

[ieag051-B21] Hinton HE. 1981. Defensive devices. In: Biology of insect eggs. 1st ed. Pergamon Press. p. 240–268. 10.1016/b978-1-4832-8401-9.50015-5

[ieag051-B22] Hoang DT , Chernomor O, von Haeseler A, et al 2018. UFBoot2: improving the ultrafast bootstrap approximation. Mol. Biol. Evol. 35:518–522. 10.1093/molbev/msx281 29077904 PMC5850222

[ieag051-B23] Kalyaanamoorthy S , Minh BQ, Wong TKF, et al 2017. ModelFinder: fast model selection for accurate phylogenetic estimates. Nat. Methods. 14:587–589. 10.1038/nmeth.4285 28481363 PMC5453245

[ieag051-B24] Kawamoto F , Kumada N. 1984. Biology and venoms of Lepidoptera. In: Tu AT, editor. Handbook of natural toxins. Vol. 2: Insect poisons, allergens, and other invertebrate venoms. Marcel Dekker. p. 291–330.

[ieag051-B25] Kemper H. 1955. Experimentelle untersuchungen über die durch afterwolle von *Euproctis chrysorrhoea* (Lepidoptera) erzeugte dermatitis, verglichen mit der wirkung von arthropodenstichen. Zeitschrift Für Angew Zool 55:37–59.

[ieag051-B26] Kobayashi H , Nonaka M. 2016. Molecular phylogeny of the Notodontidae: subfamilies inferred from 28S rRNA sequences (Lepidoptera, Noctuoidea, Notodontidae). Tinea. Suppl. 23:1–83.

[ieag051-B27] Lamy M. 1984. La processionnaire du colatier: *Anaphae venata* Butler (Lépidoptère: Thaumetopoeidae): Papillon urticant d’Afrique. Int. J. Trop. Insect Sci. 5:83–86. 10.1017/s1742758400001697

[ieag051-B28] Lundberg U , Osborn F, Carvaja Z, et al 2002. Isolation and partial characterization of a protease with kallikrein-like activity from the egg-nests of *Hylesia metabus* (Crammer 1775) (Lepidoptera: Saturnidae), preliminary communication. Rev Cient la Fac Ciencias Vet la Univ del Zulia 12:97–102.

[ieag051-B29] Mackey AP. 1985. Moth species eaten by pied butcher birds. J. Aust. Entomol. Soc. 24:93–94. 10.1111/j.1440-6055.1985.tb00194.x

[ieag051-B30] Mather A , Zalucki MP, Farrell J, et al 2019. Australian processionary caterpillars, *Ochrogaster lunifer* Herrich-Schäffer (Lepidoptera: Notodontidae), comprise cryptic species. Austral. Entomol. 58:816–825. 10.1111/aen.12410

[ieag051-B31] Mills M. 1952. Bag shelter caterpillars and their habits. West Aust. Nat. 3:84–92. 10.5962/p.312026

[ieag051-B32] Minh BQ , Nguyen MAT, von Haeseler A. 2013. Ultrafast approximation for phylogenetic bootstrap. Mol. Biol. Evol. 30:1188–1195. 10.1093/molbev/mst024 23418397 PMC3670741

[ieag051-B33] Mullen GR , Zaspel JM. 2019. Moths and butterflies (Lepidoptera). In: Mullen G, Durden L, editors. Medical and veterinary entomology. 3rd ed. Academic Press. p. 439–458. 10.1016/B978-0-12-814043-7.00021-2

[ieag051-B34] Pandey B. 2024. Morphological variations, life history and natural enemies of *Gazalina chrysolopha* (Lepidoptera: Notodontidae) in Gandaki province [masters dissertation]. Tribhuvan University, Kathmandu, Nepal. https://elibrary.tucl.edu.np/items/3b85ca4a-3467-4ce0-88cf-cc080389db55

[ieag051-B35] Perafán C , Galvis W, Gutiérrez M, et al 2016. *Kankuamo*, a new theraphosid genus from Colombia (Araneae, Mygalomorphae), with a new type of urticating setae and divergent male genitalia. Zookeys. 2016:89–109. 10.3897/zookeys.601.7704 PMC4978081 27551189

[ieag051-B36] Perkins LE , Cribb BW, Pagendam DE, et al 2019. Variation in morphology and airborne dispersal of the urticating apparatus of *Ochrogaster lunifer* (Lepidoptera: Notodontidae), an Australian processionary caterpillar, and implications for livestock and humans. J. Insect Sci. 19:1–8. 10.1093/jisesa/iez112 PMC6883397 31782508

[ieag051-B37] Perkins LE , Zalucki MP, Perkins NR, et al 2016. The urticating setae of *Ochrogaster lunifer*, an Australian processionary caterpillar of veterinary importance. Med. Vet. Entomol. 30:241–245. 10.1111/mve.12156 26669823

[ieag051-B38] Pesce H , Delgado A. 1971. Poisoning from adult moths and caterpillars. In: Bücherl W, Buckley EE, editors. Venomous animals and their venoms, Volume III Venomous invertebrates. Academic Press. p. 119–156.

[ieag051-B39] Petrucco Toffolo E , Zovi D, Perin C, et al 2014. Size and dispersion of urticating setae in three species of processionary moths. Integr. Zool. 9:320–327. 10.1111/1749-4877.12031 24952969

[ieag051-B40] Pomeroy AWJ. 1921. The irritating hairs of the wild silk moths of Nigeria. Bull. Imp. Inst. 19:311–319. 10.1542/peds.17.2.278

[ieag051-B41] Regier JC , Mitter C, Mitter K, et al 2016. Further progress on the phylogeny of Noctuoidea (Insecta: Lepidoptera) using an expanded gene sample. Syst. Entomol. 42:82–93. 10.1111/syen.12199

[ieag051-B42] Rodriguez J , Vicente Hernández J, Fornés L, et al 2004. External morphology of abdominal setae from male and female *Hylesia metabus* adults (Lepidoptera: Saturniidae) and their function. Florida Entomol. 87:30–36. 10.1653/0015-4040(2004)087[0030:EMOASF]2.0.CO;2

[ieag051-B43] Rothschild M , Reichstein T, von Euw J, et al 1970. Toxic Lepidoptera. Toxicon 8:293–299. 10.1016/0041-0101(70)90006-1 5531242

[ieag051-B44] Southcott RV. 1987. Moths and butterflies. In: Covacevich P, Davie P, Pearn J, editors. Toxic plants and animals. A guide for Australia. Queensland Museum. p. 242–257.

[ieag051-B45] St Laurent RA , Goldstein L, Prada-Lara L, et al 2025. Phylogenomics of prominent moths (Lepidoptera: Notodontidae): a subfamily‑level reclassification. Smithson Contrib to Zool 657:1–133.

[ieag051-B46] Tsutsumi C. 1958. A histological study of the development of the urticating spicules of the far eastern urticating moth, *Euproctis flava* Bremer (Lepidoptera: Lymantriidae). Jpn. J. Med. Sci. Biol. 11:443–453. 10.7883/yoken1952.11.443 13653847

[ieag051-B47] Upadhyay MP , Shrestha BR. 2017. SHAPU: forty years on mystery persists. Nepal J. Ophthalmol. 9:13–16. 10.3126/nepjoph.v9i1.17527 29022949

[ieag051-B48] Werno J , Lamy M. 1990. Pollution atmospherique d’origine animale: Les poils urticants de la chenille processionnaire du pin (*Thaumetopoea pityocampa* Schiff.) (Insectes, Lepidopteres). Comptes Rendus l’Academie des Sci—Ser III 310:325–331. 2111200

[ieag051-B49] Werno J , Lamy M. 1994. Daily cycles for emission of urticating hairs from the pine processionary caterpillar (*Thaumetopoea pityocampa* S.) and the brown tail moth (*Euproctis chrysorrhoea* L) (Lepidoptera) in laboratory conditions. Aerobiologia (Bologna) 10:147–151. 10.1007/BF02459229

[ieag051-B50] Wirtz RA. 1984. Allergic and toxic reactions to non-stinging arthropods. Annu. Rev. Entomol. 29:47–69. 10.1146/annurev.en.29.010184.000403 6362550

[ieag051-B51] Wong T , Ly-Trong N, Ren H, et al. 2026. IQ-TREE 3: phylogenomic inference software using complex evolutionary models. Mol. Biol. Evol. 43. 10.1093/MOLBEV/MSAG117 PMC13191116 42085559

