# Supplementary material for: Armoured Amazon female moths: urticating setae in Notodontidae (Lepidoptera)
Source: J Insect Sci. 2026 Jul 2;26(4):ieag051. doi: 10.1093/jisesa/ieag051 (PMC13326758; doi:10.1093/jisesa/ieag051)
Supplement: ieag051_Supplementary_Data [file ieag051_supplementary_data.zip › Supplementary Figure S1.docx]

| **Species** | **Dorsal** | **Corethrogyne** |
| --- | --- | --- |
| **Anaphinae** | | |
| *Adrallia bipunctata* | 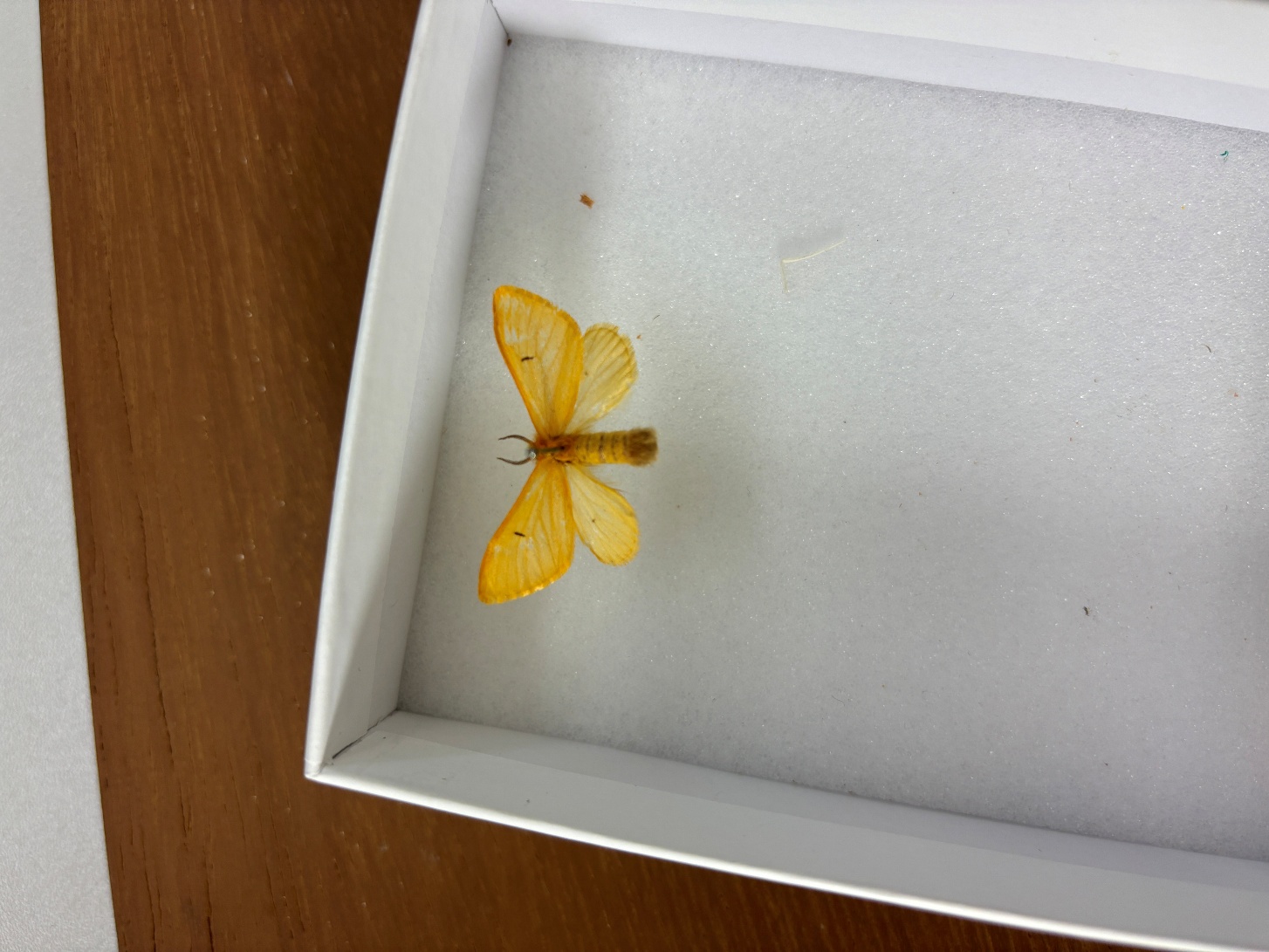 | 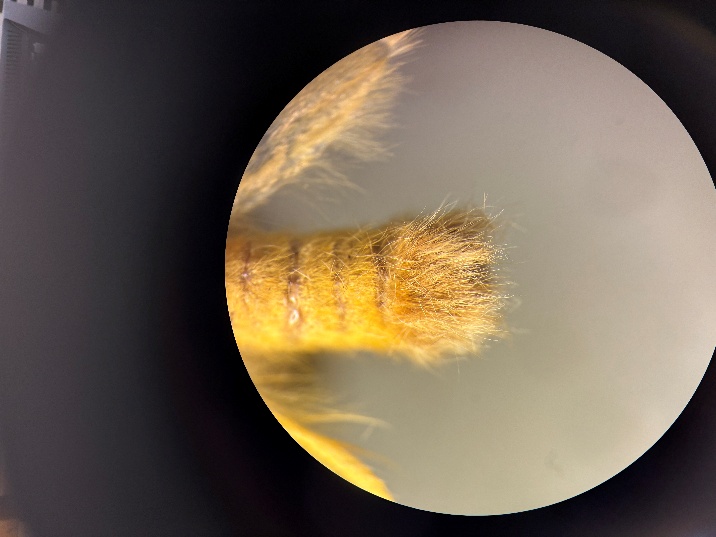 |
| *Anaphe panda* | 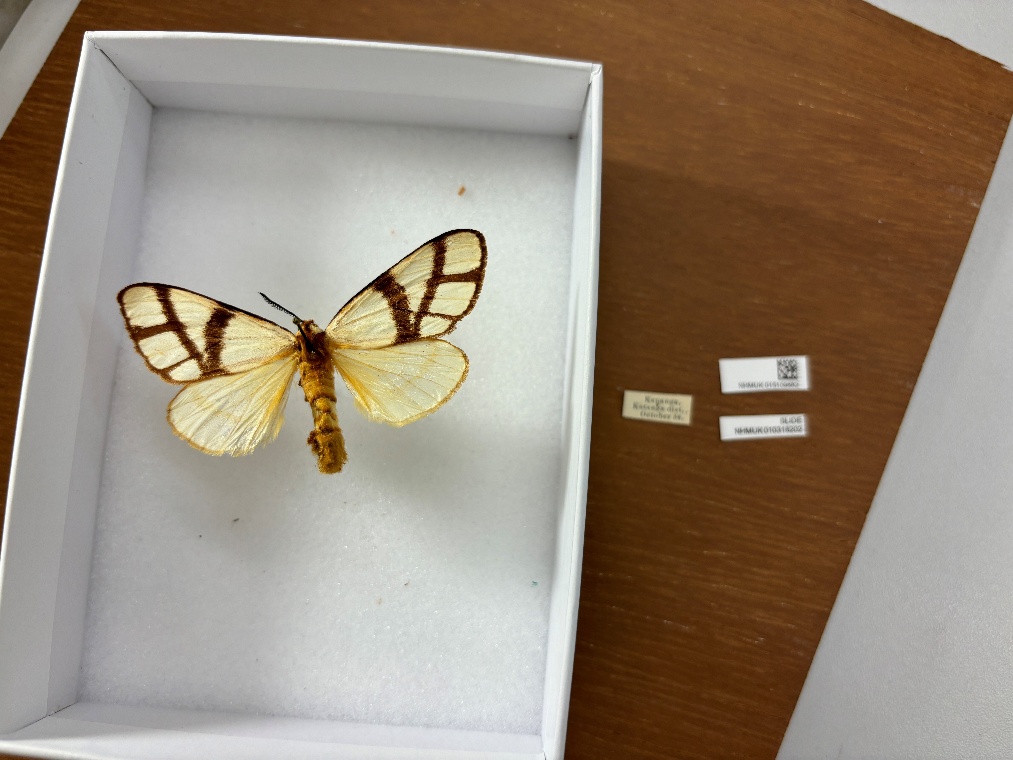 | 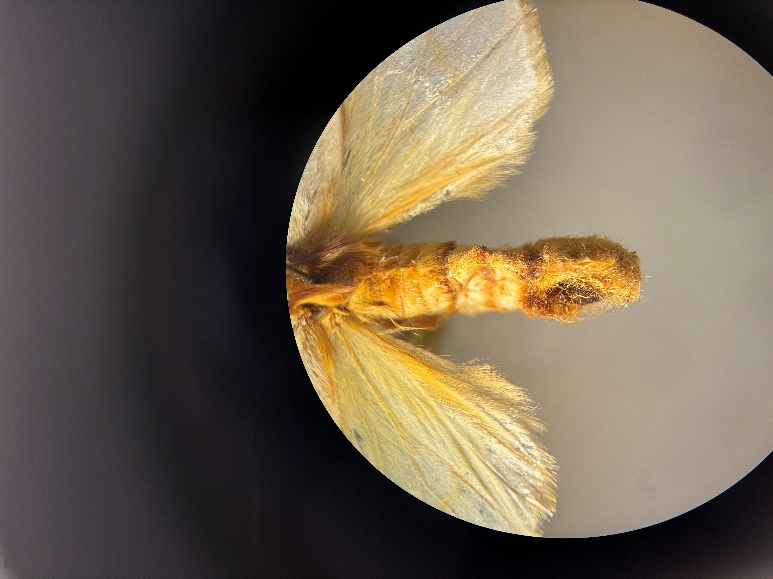 |
| *Anaphe reticulata* | 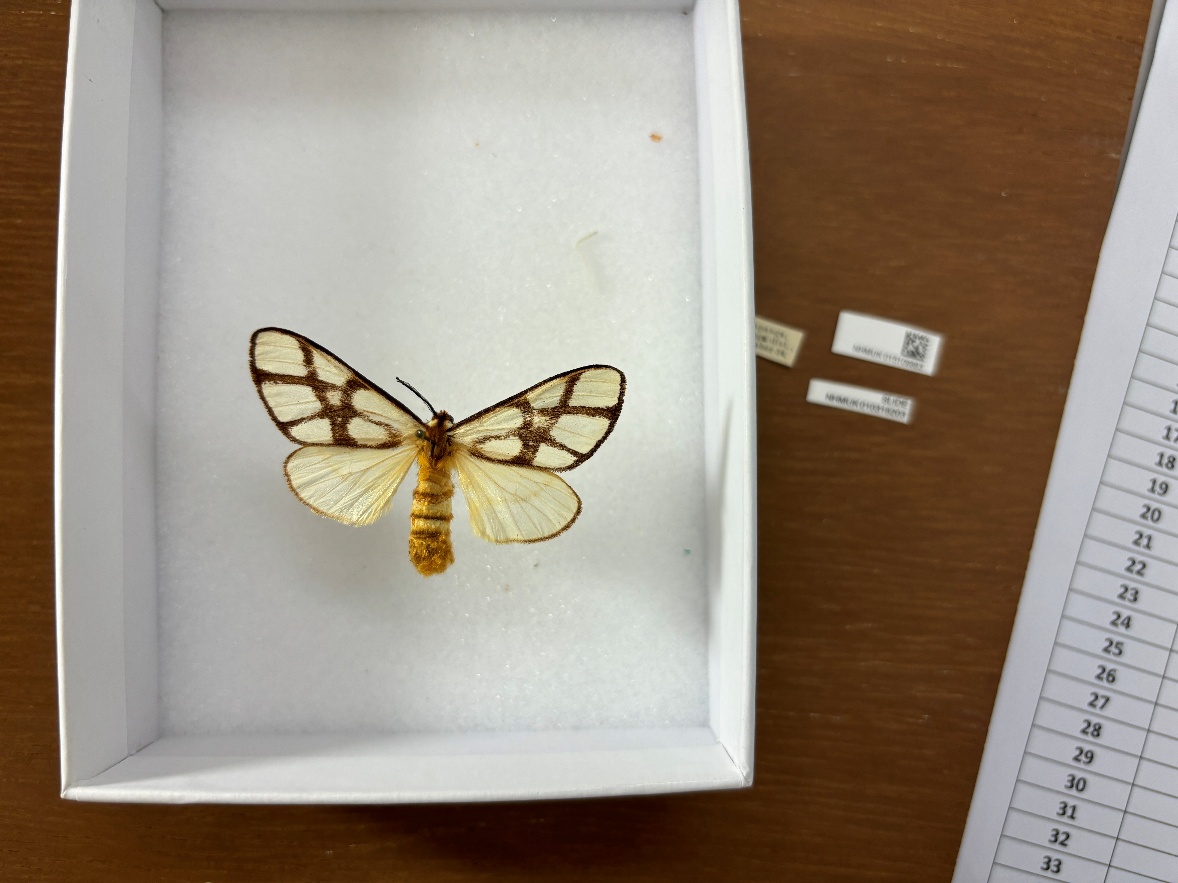 | 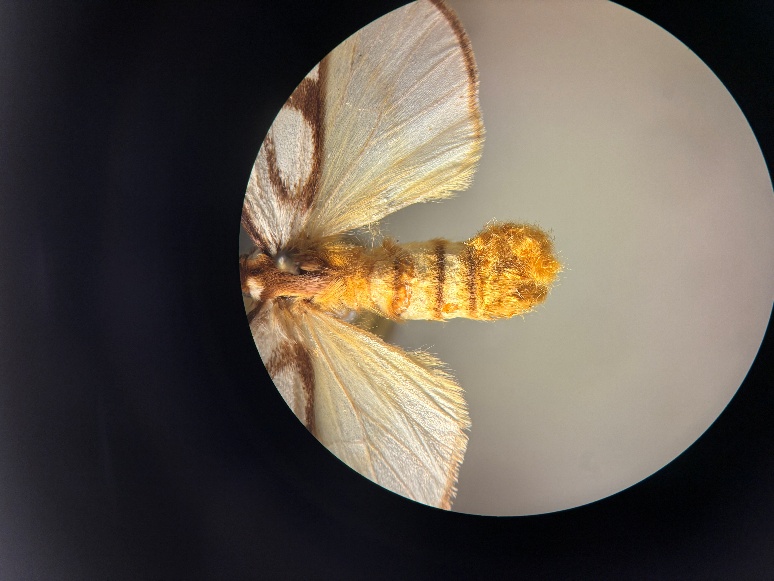 |
| *Anaphe venata* | 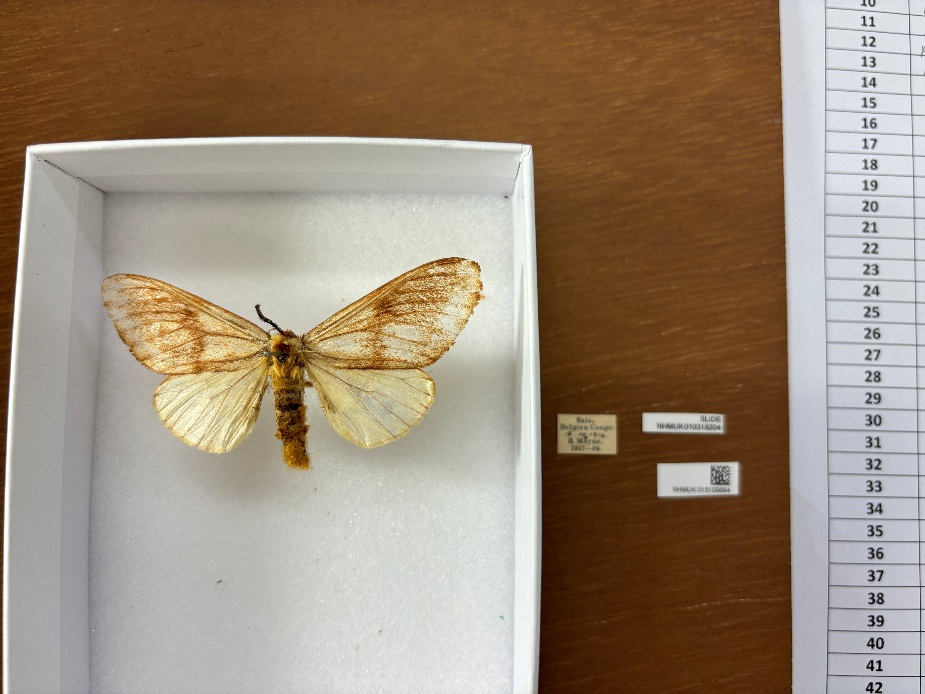 | 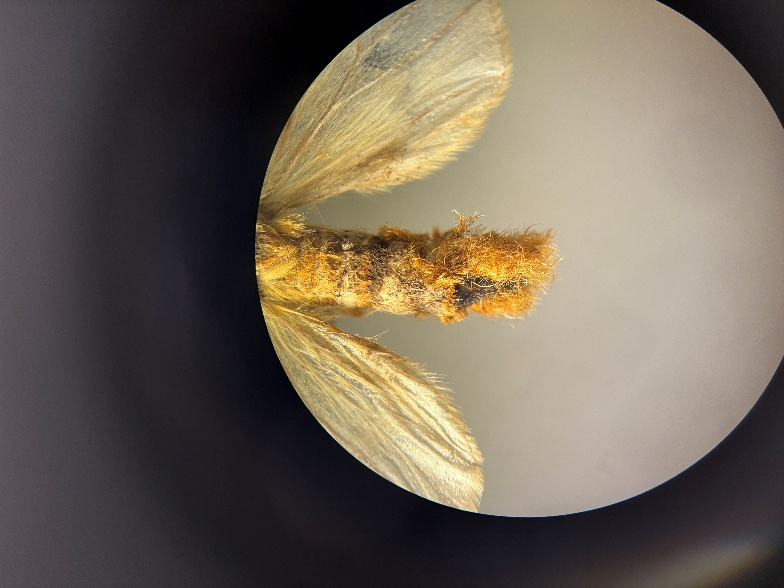 |
| *Epanaphe moloneyi* | 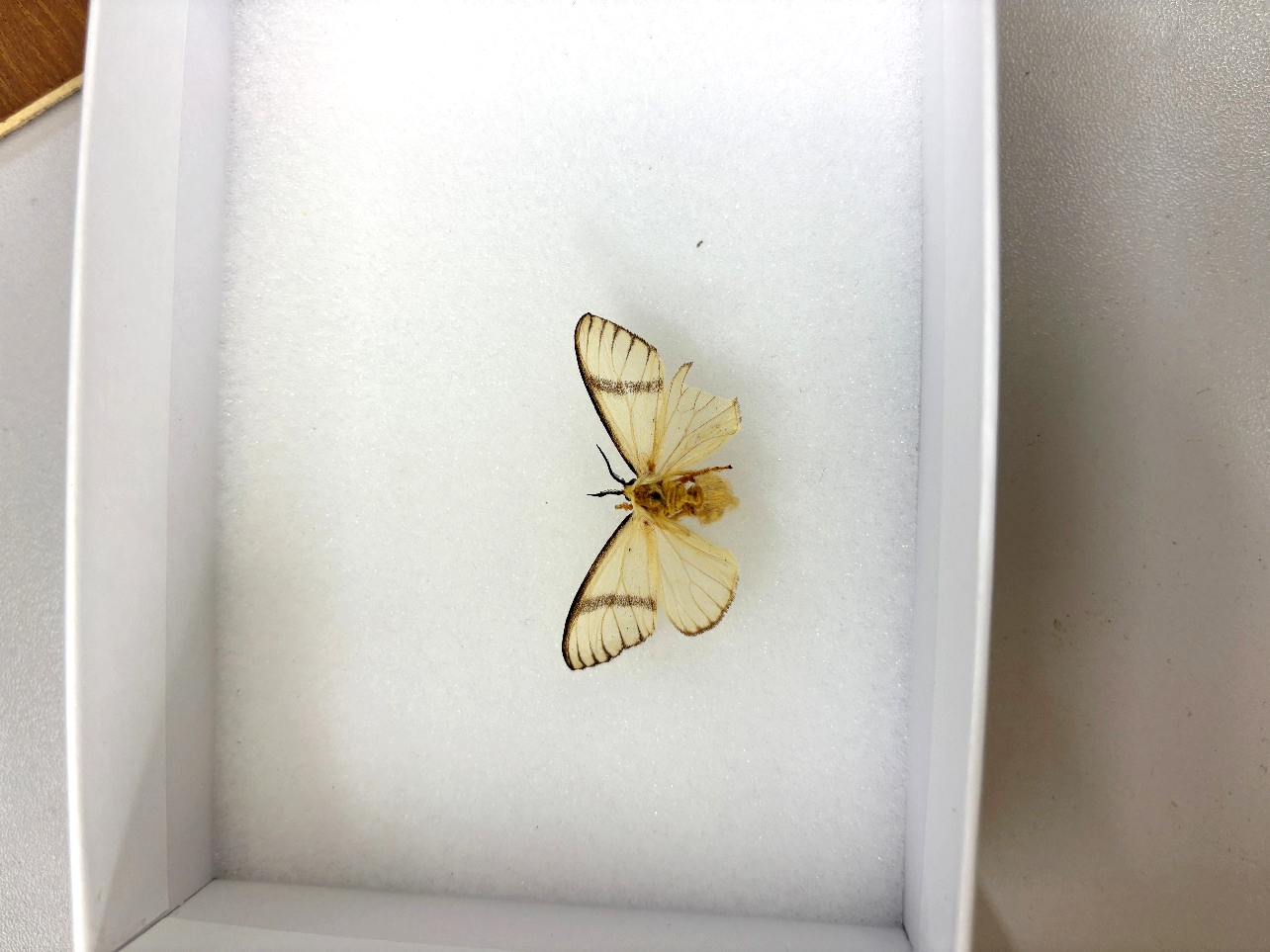 | 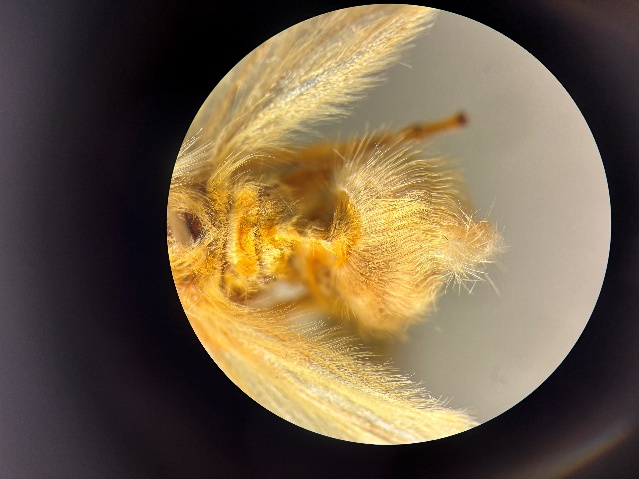 |
| *Hypsoides bipars* | 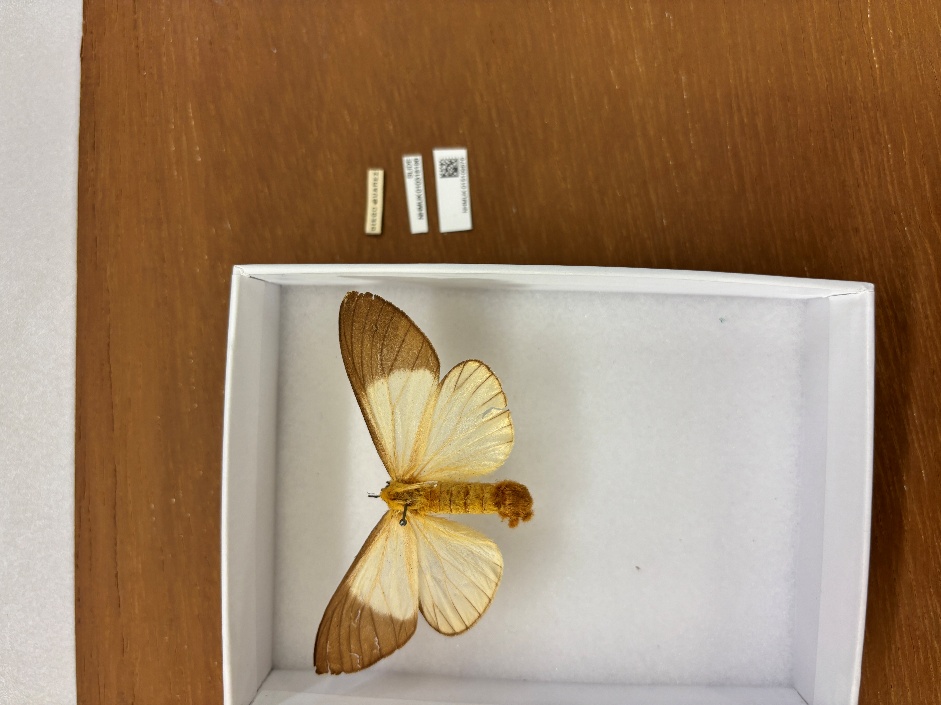 | 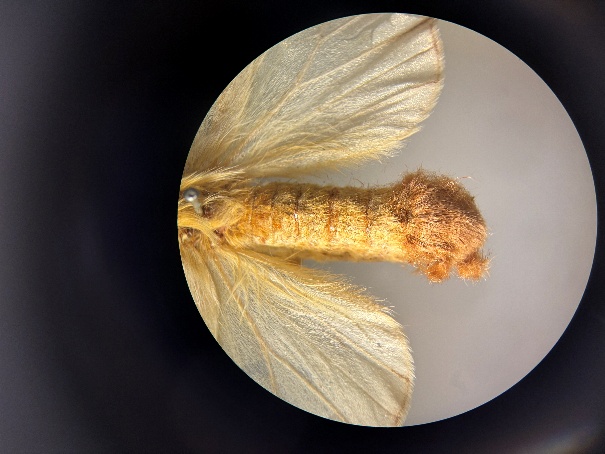 |
| *Hypsoides* cf. *meloui* | 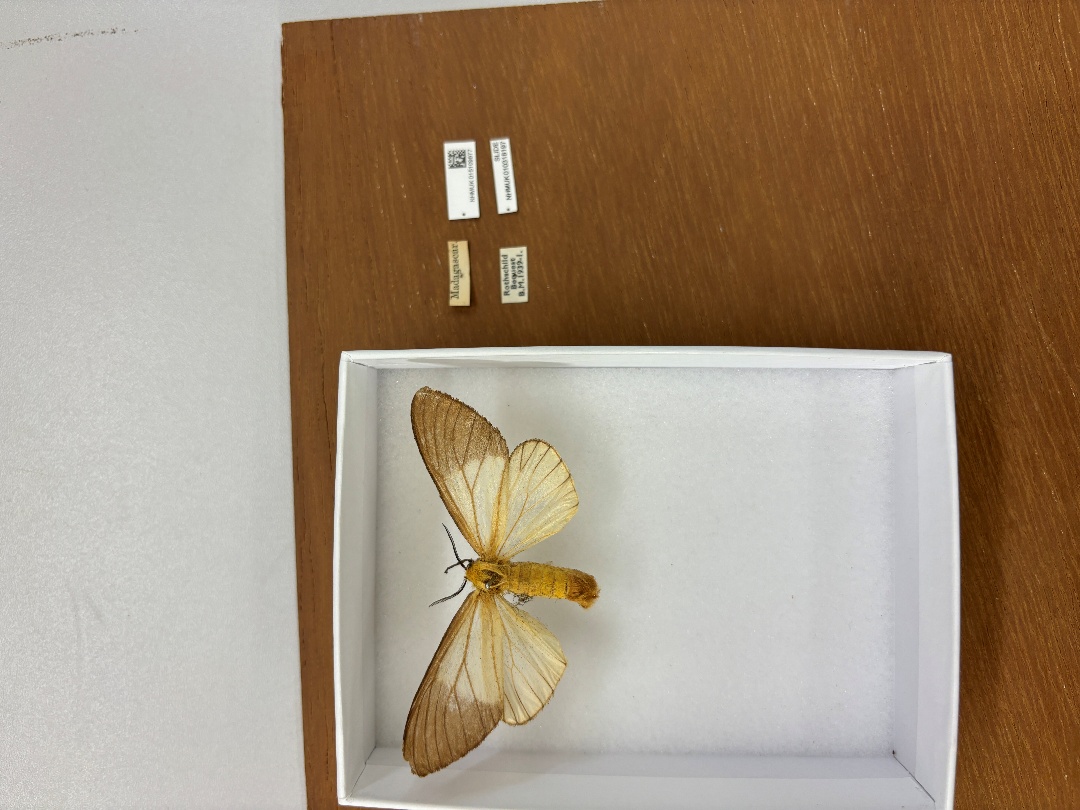 | 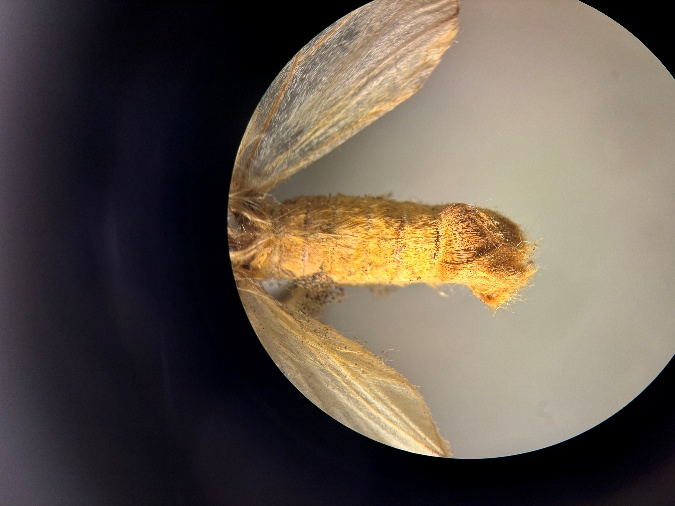 |
| *Paradrallia punctigera* | 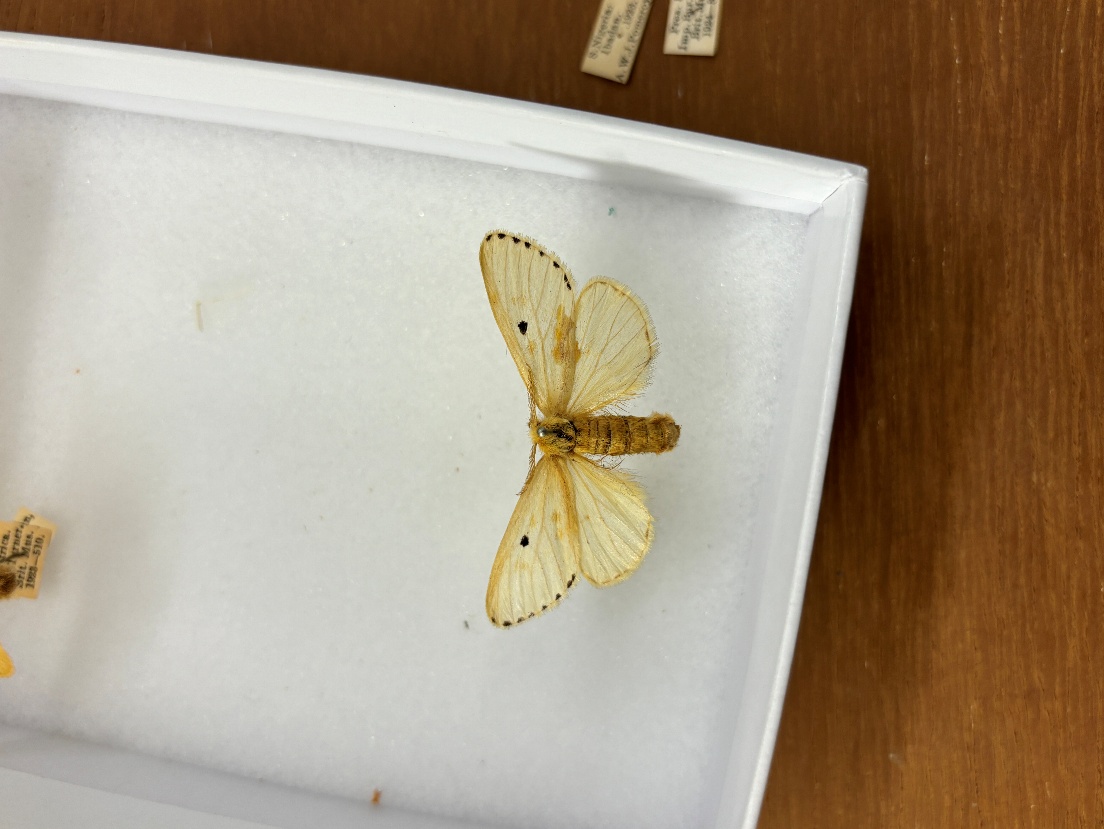 | 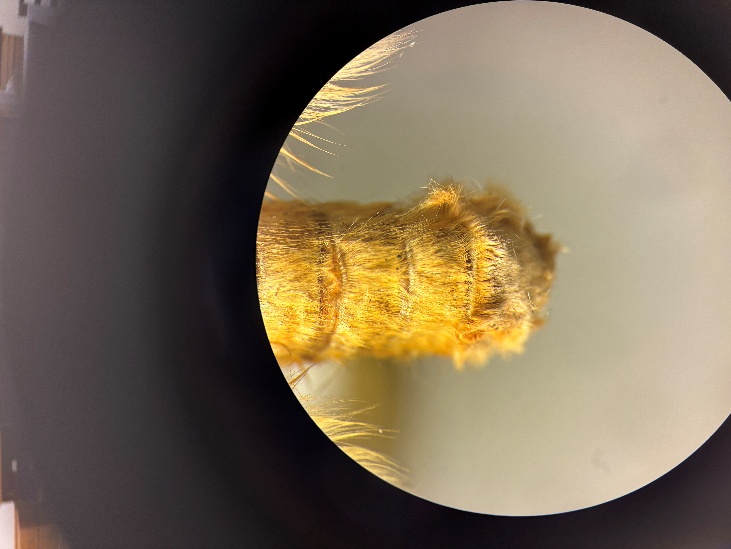 |
| **Thaumetopoeinae** | | |
| *Epicoma argentata* | 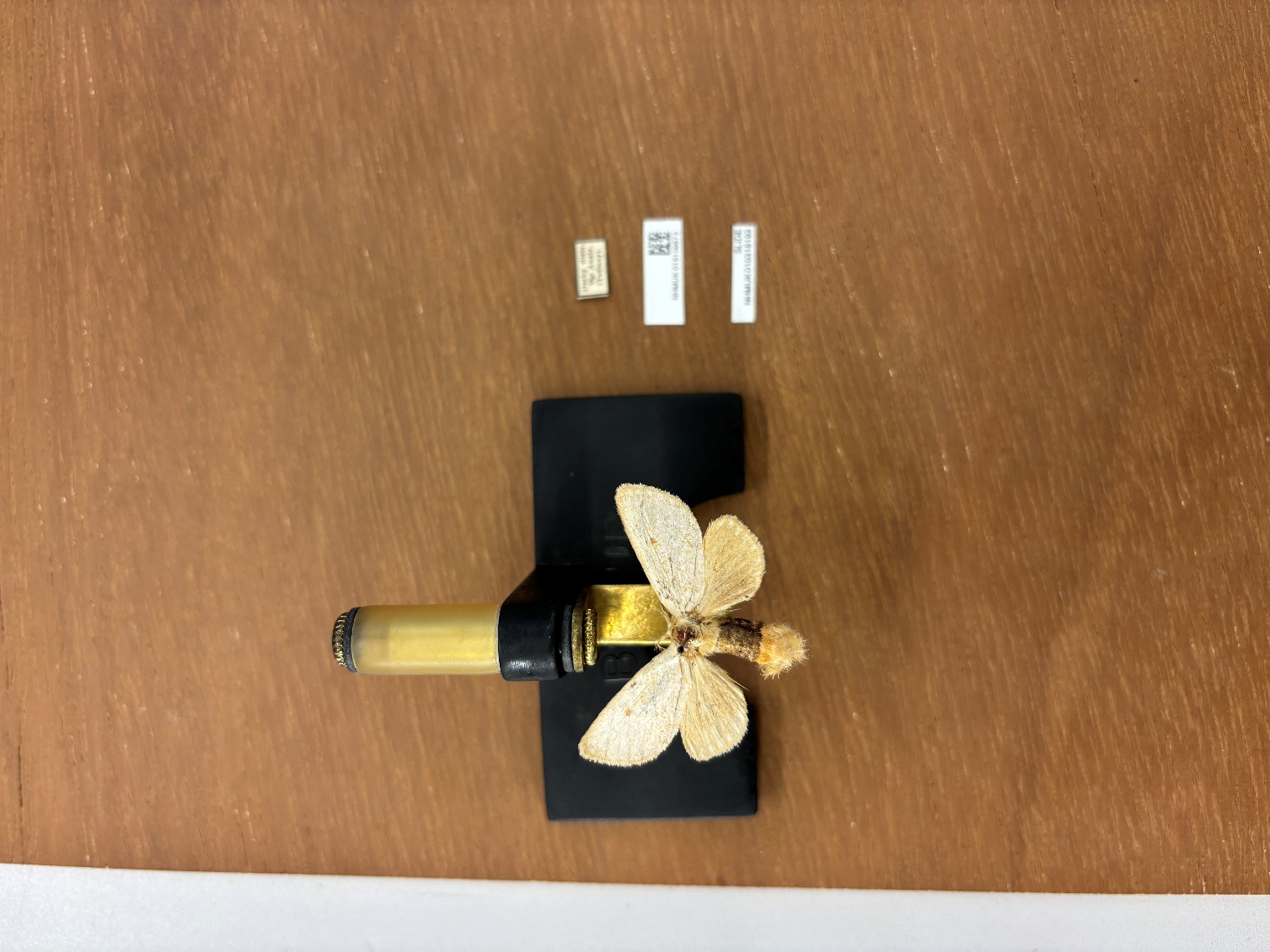 | 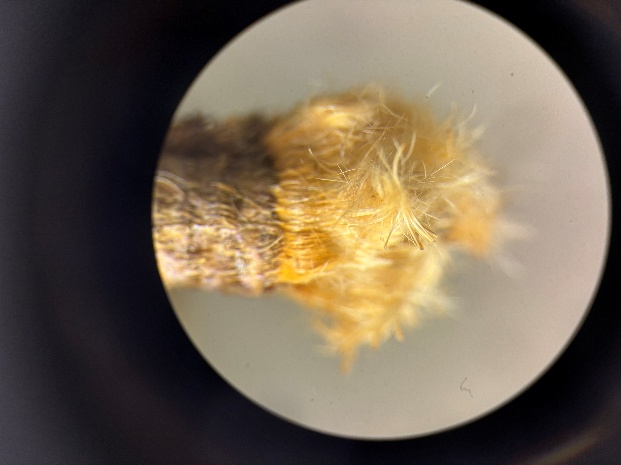 |
| *Epicoma signata* | 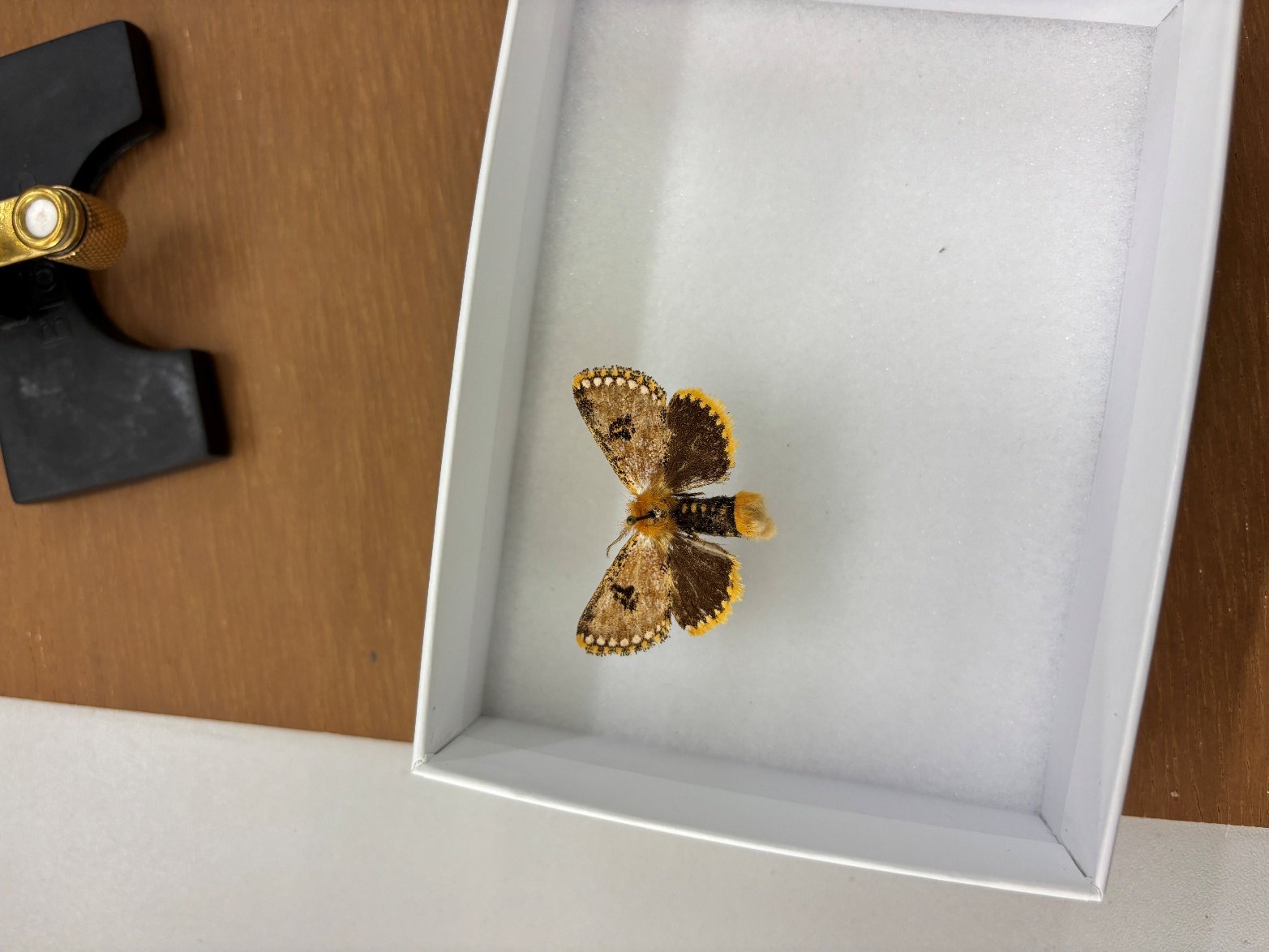 | 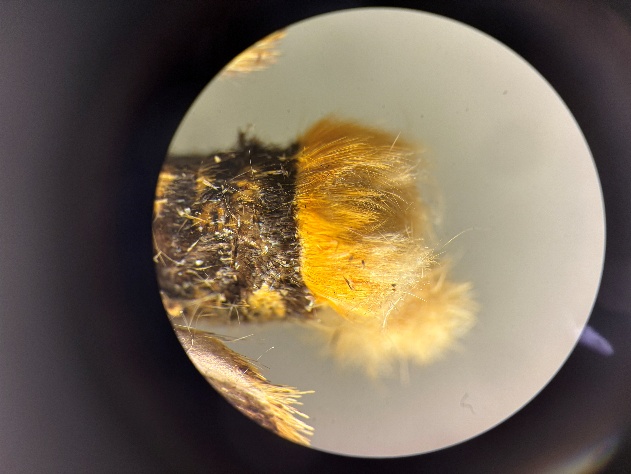 |
| *Gazalina chrysolopha* | 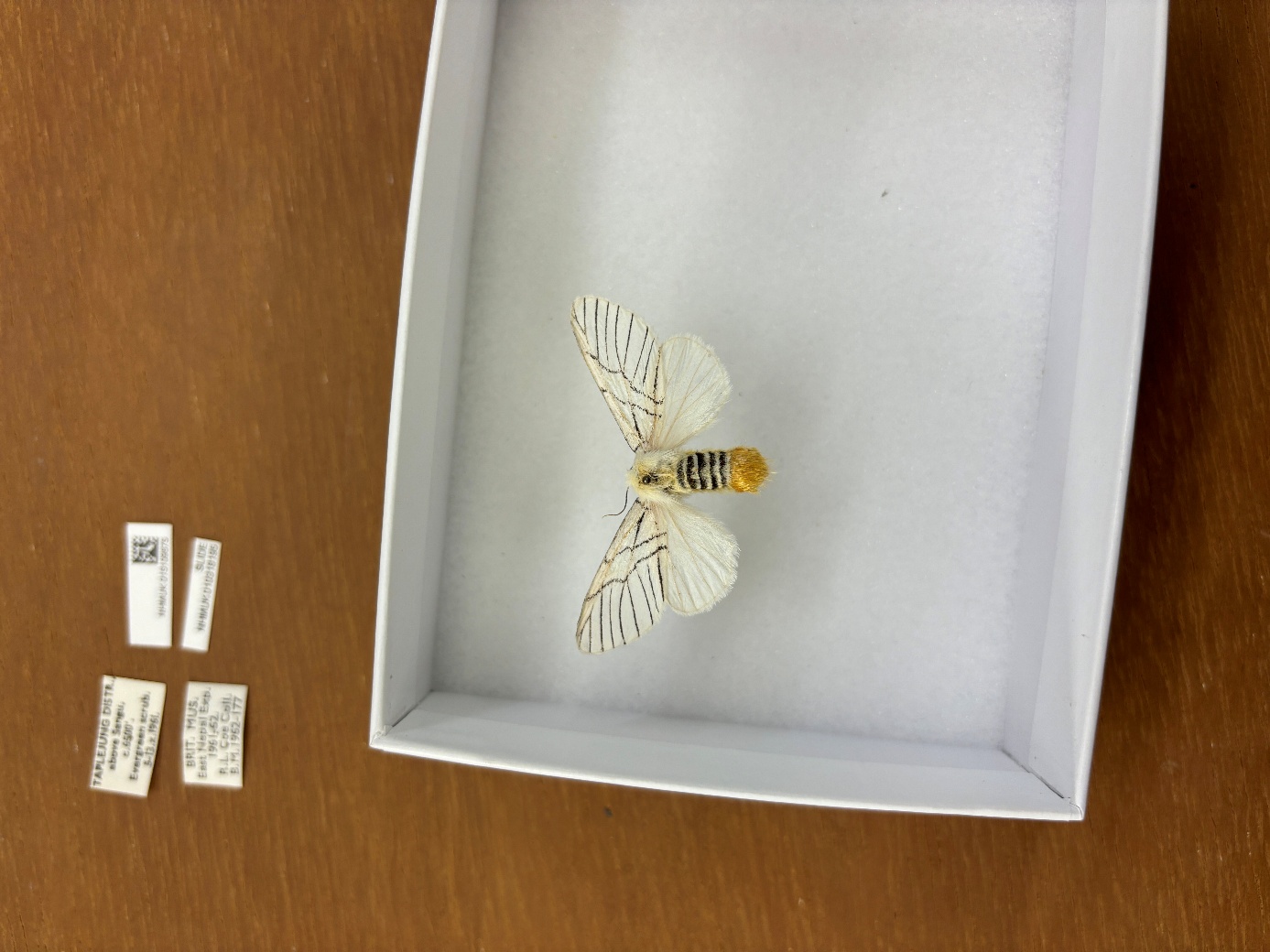 | 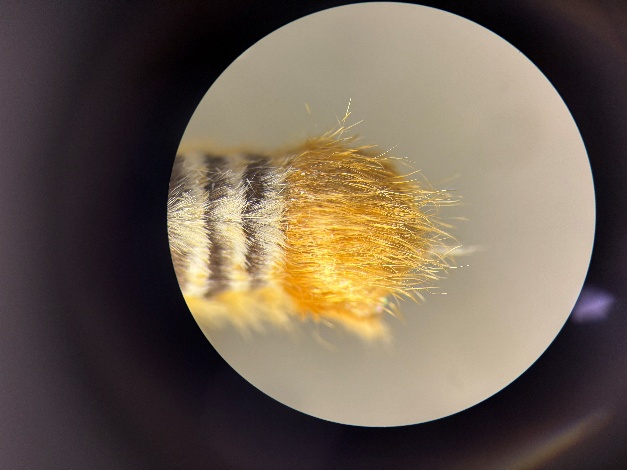 |
|  |  |  |
|  |  |  |
| *Ochrogaster lunifer* | 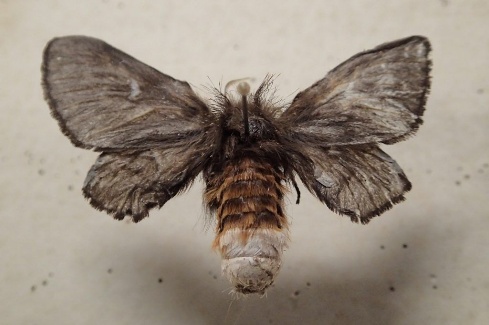 | 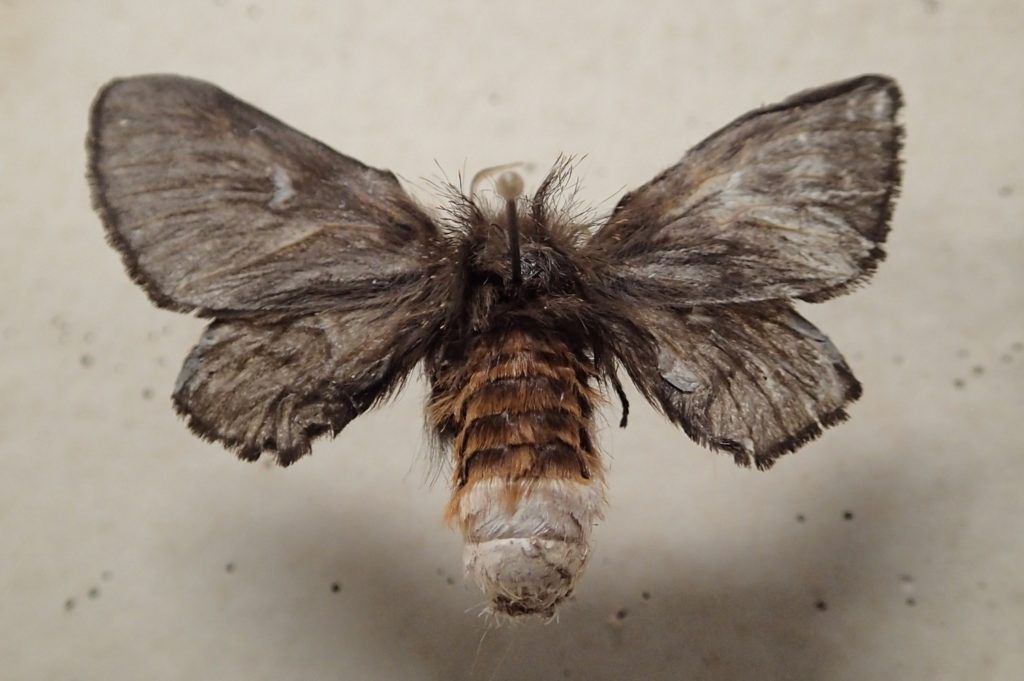 |
| *Tanystola isabella* | 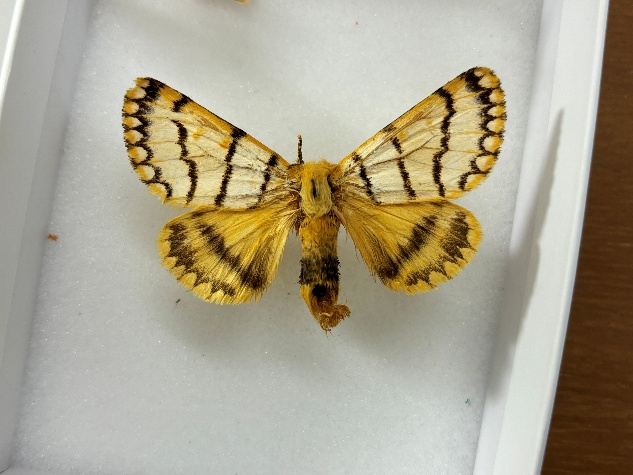 | 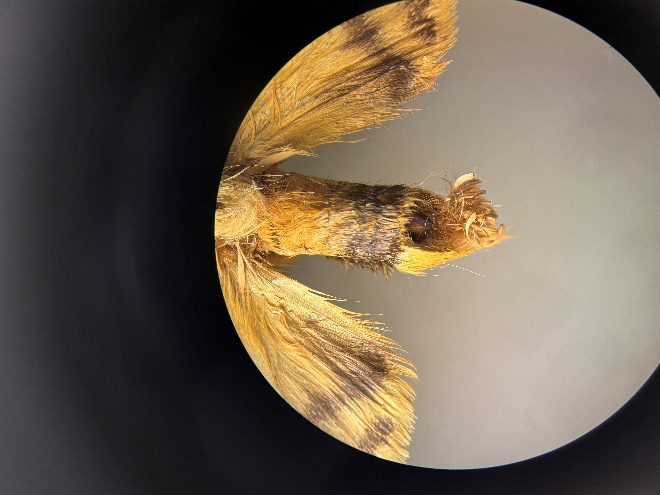 |
| *Trichiocercus sparshalli* | 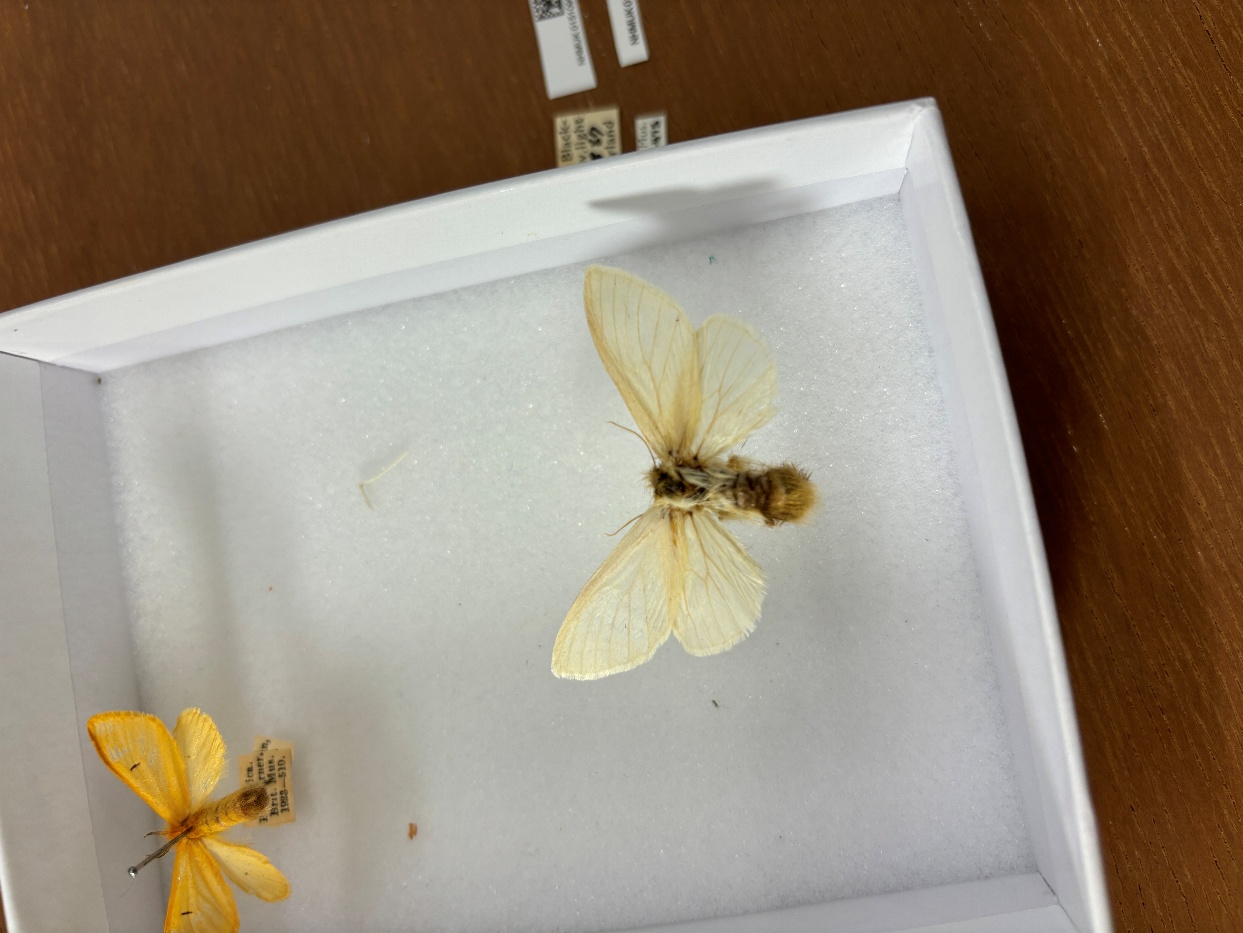 | 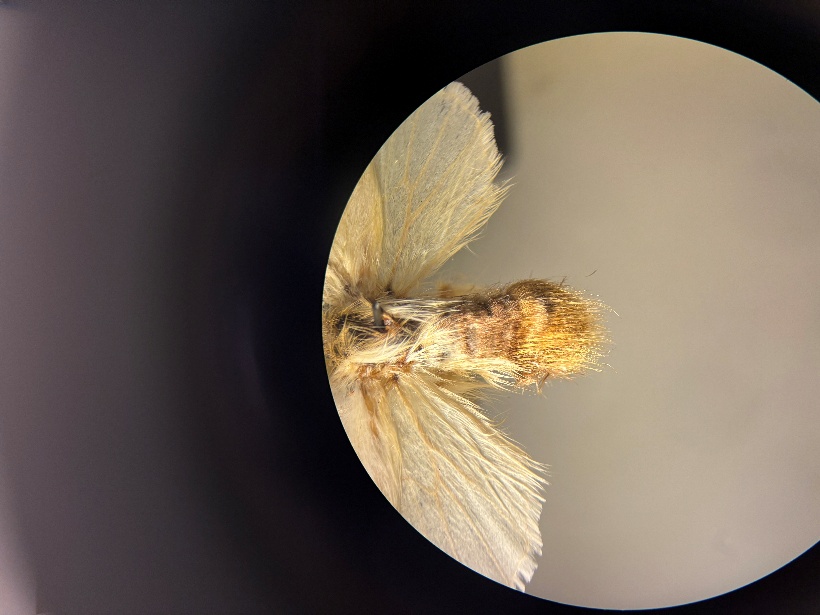 |

**Supplementary Fig. S1.** All the Notodontidae specimens used in this study from the British Natural History Museum and *Ochrogaster lunifer* from The University of Queensland. Scale bar not provided. Images taken with an iPhone 15, close up of the posterior body was taken through the eyepiece of a stereomicroscope.
