# Supplementary material for: Armoured Amazon female moths: urticating setae in Notodontidae (Lepidoptera)
Source: J Insect Sci. 2026 Jul 2;26(4):ieag051. doi: 10.1093/jisesa/ieag051 (PMC13326758; doi:10.1093/jisesa/ieag051)
Supplement: ieag051_Supplementary_Data [file ieag051_supplementary_data.zip › Supplementary Figure S2.pdf]

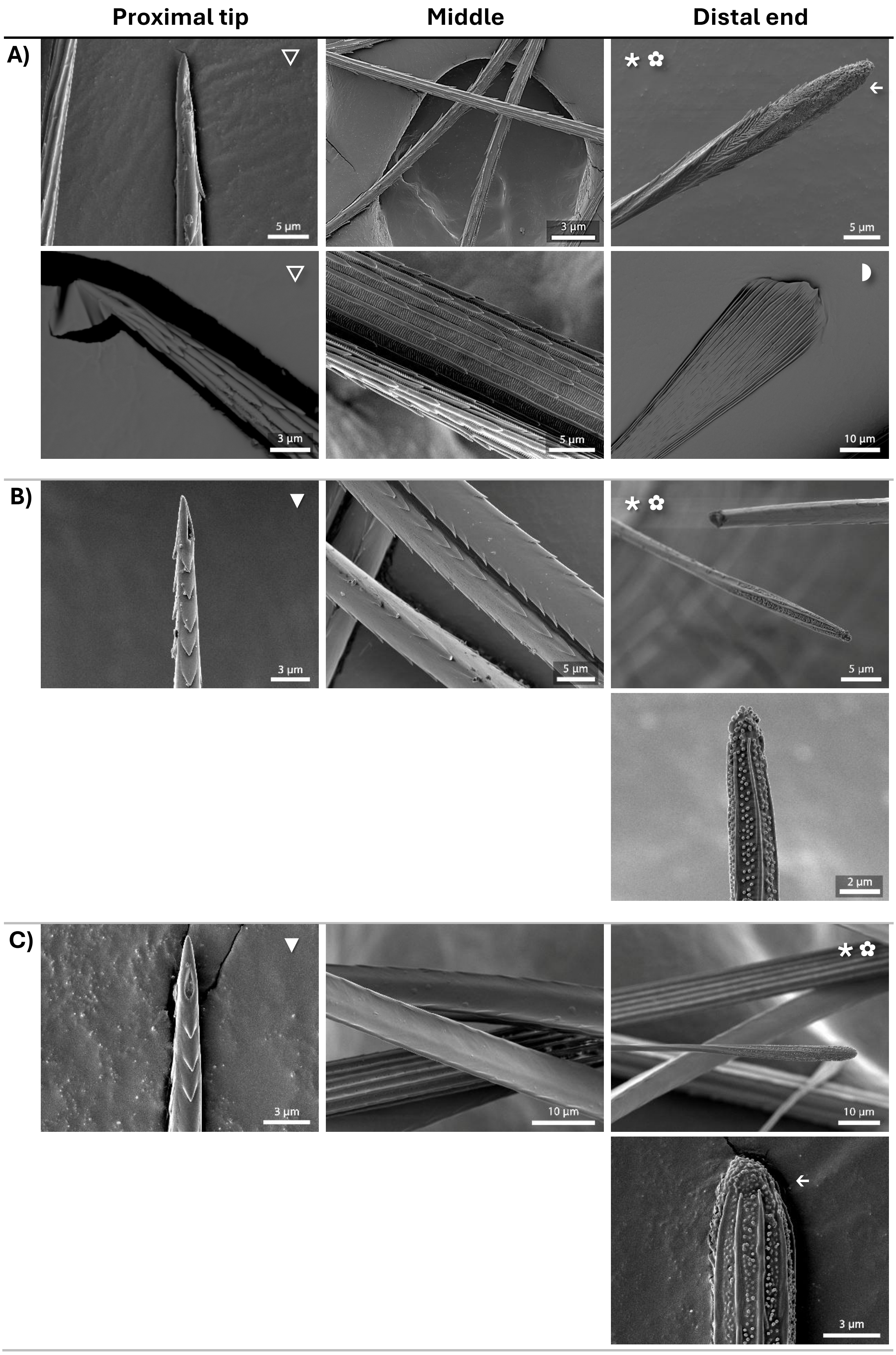
Proximal tip
Middle
Distal end
A)
5 µm
3 µm
5 µm
3 µm
5 µm
10 µm
B)
3 µm
5 µm
5 µm
2 µm
C)
3 µm
10 µm
10 µm
3 µm

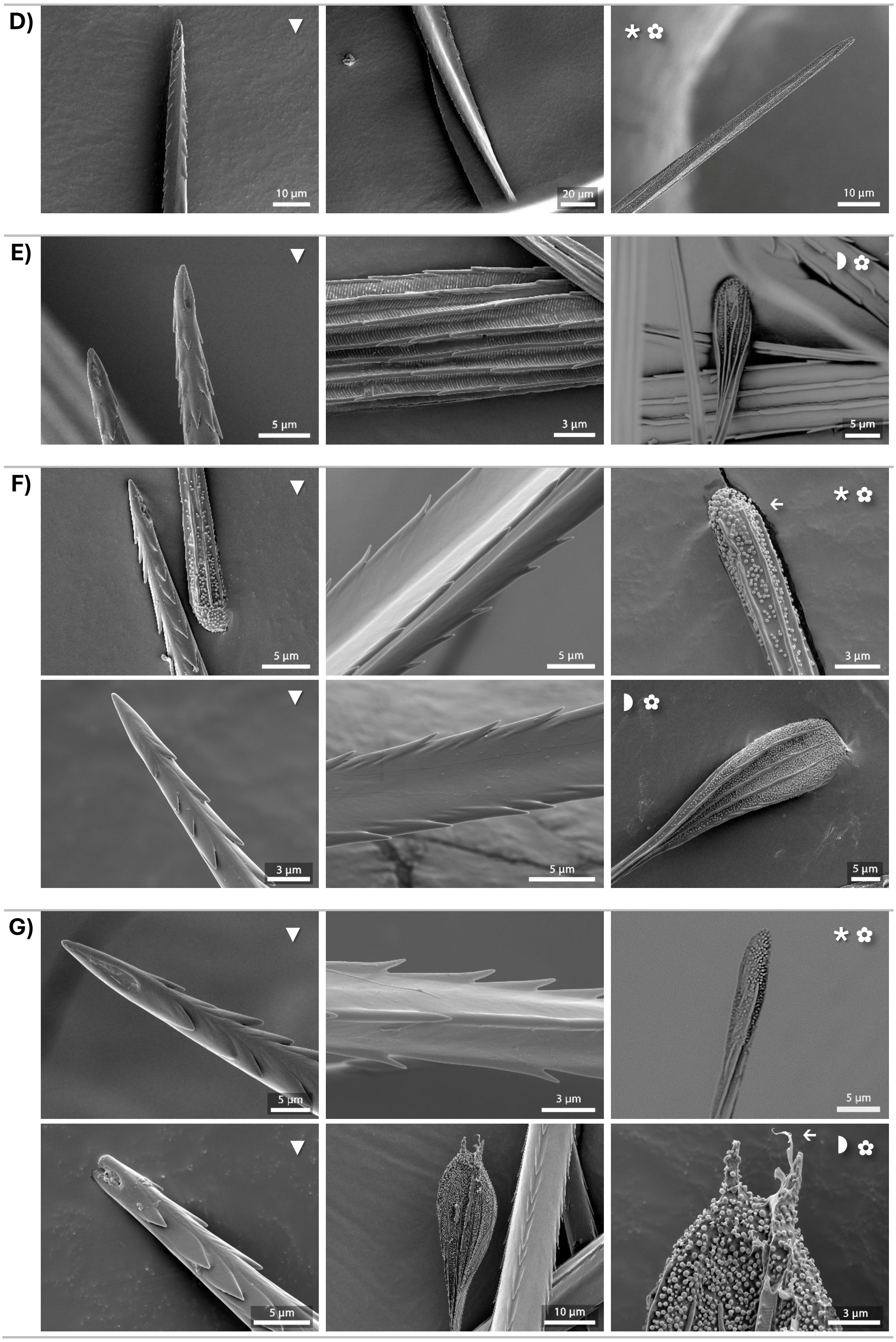
D)
10 µm
20 µm
10 µm
E)
5 µm
3 µm
5 µm
F)
5 µm
5 µm
3 µm
3 µm
5 µm
5 µm
G)
5 µm
3 µm
5 µm
5 µm
10 µm
3 µm

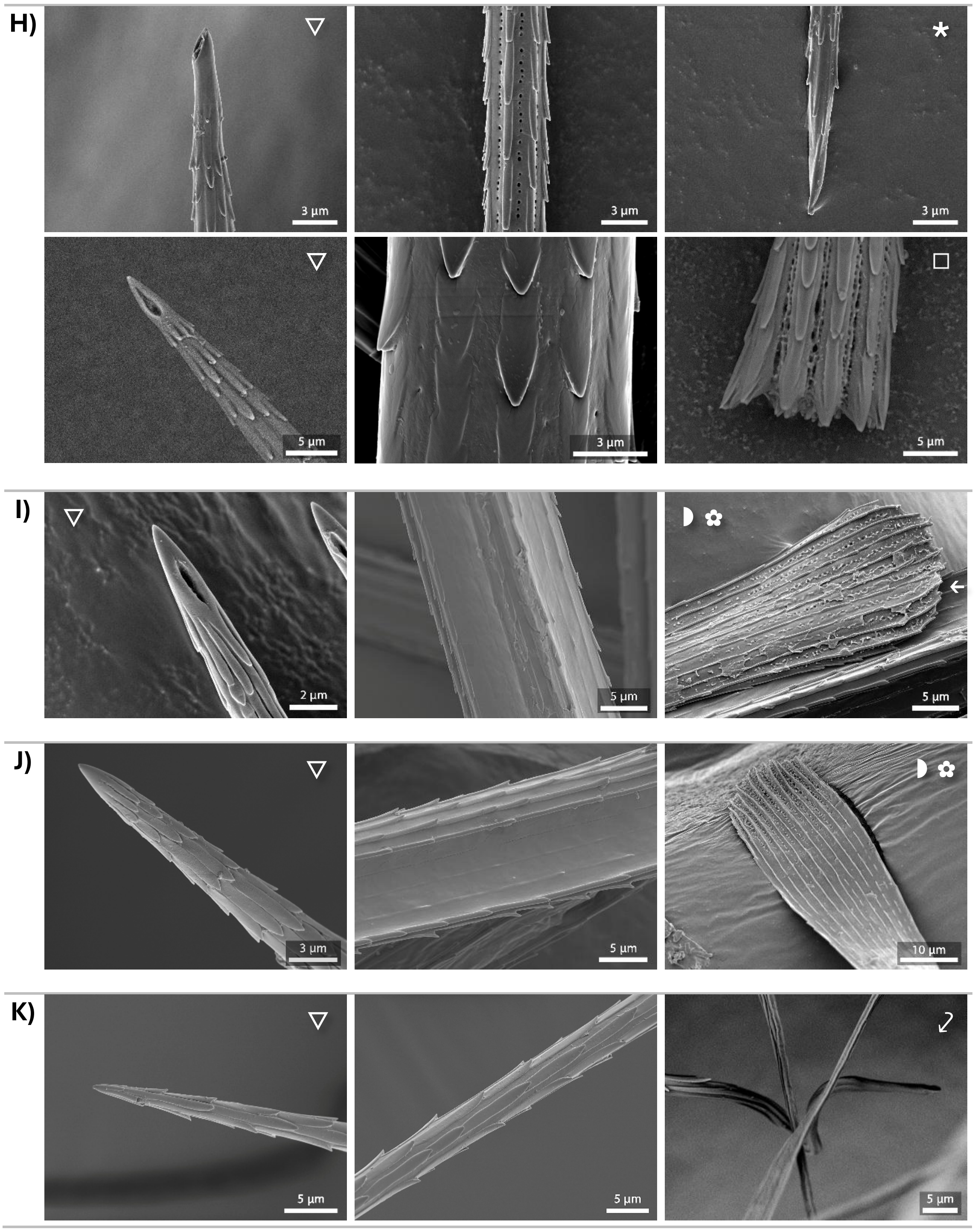
H)
3 µm
3 µm
3 µm
5 µm
3 µm
5 µm
I)
2 µm
5 µm
5 µm
J)
3 µm
5 µm
10 µm
K)
5 µm
5 µm
5 µm

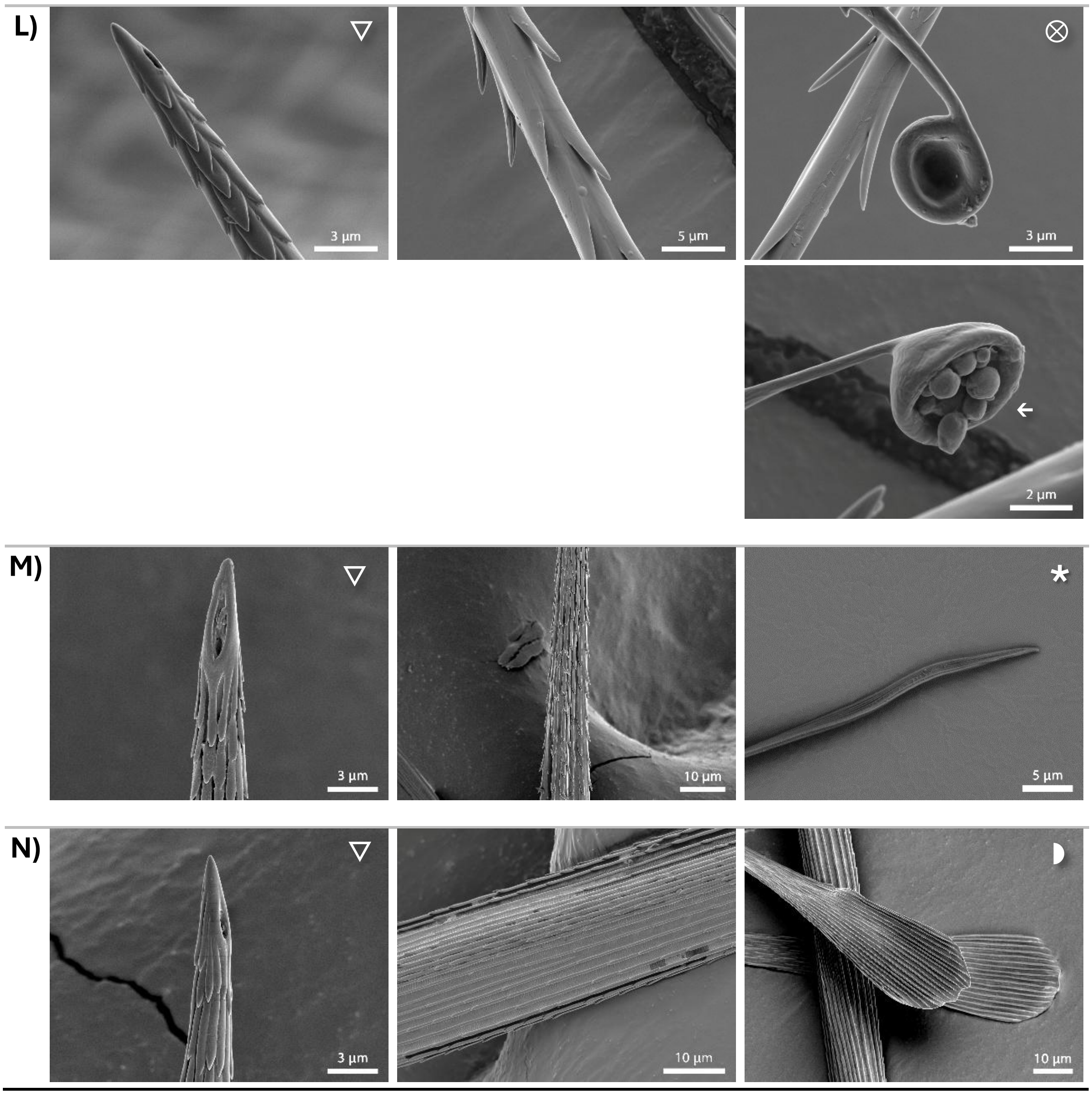

Supplementary Fig. S2. Scanning electron microscopy (SEM) images of true setae from the female corethrogyne of 14 species of Notodontidae. Left column: Proximal tip of the seta (▽ lobed or ▼ serrate) where it previously attached into a socket on the last abdominal segment. Middle column: Midpoint of the seta. Right column: Distal end of the seta (* tapered, ◗ spatulate, ☐ blunt. ⤦ hook, ⊗ cup, ✿ verrucose and ← secretion). Anaphinae: A) *Adrallia bipunctata*, B) *Anaphe panda*, C) *An. reticulata*, D) *An. venata*, E) *Epanaphe moloneyi*, F) *Hypsoides bipars*, G) *H.* cf. *meloui* and H) *Paradrallia punctigera*. Thaumetopoeinae: I) *Epicoma argentata*, J) *Epi. signata*, K) *Gazalina chrysolopha*, L) *Ochrogaster lunifer,* M) *Tanystola isabella* and N) *Trichiocercus sparshalli*. SEM images of the distal end of the setae key morphological characteristics in selected taxa.
