# Supplementary material for: Armoured Amazon female moths: urticating setae in Notodontidae (Lepidoptera)
Source: J Insect Sci. 2026 Jul 2;26(4):ieag051. doi: 10.1093/jisesa/ieag051 (PMC13326758; doi:10.1093/jisesa/ieag051)
Supplement: ieag051_Supplementary_Data [file ieag051_supplementary_data.zip › Supplementary Table S1.pdf]

**Table S1.** BOLD reference sequences used to construct phylogenetic tree in Fig. S2

**TableS1**

| Specimens ID | BIN BOLD | Genus | Species | Identification Rank | Country | Biogeographic realms |
|---|---|---|---|---|---|---|
| ANICD110-10 | BOLD:AAF7420 | Aglaosoma | *Aglaosoma periblepta* | species | AU | Australasia |
| GWOR3089-08 | BOLD:AAF7420 | Aglaosoma | *Aglaosoma periblepta* | species | AU | Australasia |
| AMUB456-11 | BOLD:ABY7392 | Aglaosoma | *Aglaosoma variegata* | species | AU | Australasia |
| ANICD038-10 | BOLD:AAL6444 | Aglaosoma | *Aglaosoma variegata* | species | AU | Australasia |
| GWORD071-07 | BOLD:AAJ1215 | Aglaosoma | *Aglaosoma variegata* | species | AU | Australasia |
| NSWHP5572-19 | BOLD:ABY7392 | Aglaosoma | *Aglaosoma variegata* | species | AU | Australasia |
| GWOUF368-20 | BOLD:AEE1092 | Anaphe | *Anaphe* sp. | genus | ZA | Afrotropic |
| GWOUF888-20 | BOLD:AEE0747 | Anaphe | *Anaphe* sp. | genus | ZA | Afrotropic |
| KINS001-10 | BOLD:AAH6113 | Anaphe | *Anaphe panda* | species | KE | Afrotropic |
| ANLMN4585-21 | BOLD:AEE1092 | Anaphe | *Anaphe reticulata* | species | MZ | Afrotropic |
| GBAAM2406-25 | BOLD:ABA6596 | Anaphe | *Anaphe venata* | species | CM | Afrotropic |
| GBGL38570-19 | BOLD:AEI3868 | Anaphe | *Anaphe venata* | species | AO | Afrotropic |
| ANICD121-10 | BOLD:AAO0906 | Axiocleta | *Axiocleta perisema* | species | AU | Australasia |
| ANICD124-10 | BOLD:AAO6993 | Axiocleta | *Axiocleta perisema* | species | AU | Australasia |
| ANIC432-06 | BOLD:AAD5304 | Cynosarga | *Cynosarga ornata* | species | AU | Australasia |
| MGABA054-10 | BOLD:AAI5254 | Epanaphe | *Epanaphe* sp. | genus | GA | Afrotropic |
| MGABA714-10 | BOLD:AAH6188 | Epanaphe | *Epanaphe* sp. | genus | GA | Afrotropic |
| MGABB821-10 | BOLD:AAL8731 | Epanaphe | *Epanaphe* sp. | genus | GA | Afrotropic |
| MGABB927-10 | BOLD:AAL8755 | Epanaphe | *Epanaphe* sp. | genus | GA | Afrotropic |
| MGABC029-10 | BOLD:AAH6374 | Epanaphe | *Epanaphe* sp. | genus | GA | Afrotropic |
| LTOLB149-08 | BOLD:AAH6075 | Epanaphe | *Epanaphe carteri* | species | CD | Afrotropic |
| GWOSV354-11 | BOLD:ABU6548 | Epanaphe | *Epanaphe clarilla* | species | GH | Afrotropic |
| PMANL100-08 | BOLD:AAF0246 | Epanaphe | *Epanaphe moloneyi* | species | NG | Afrotropic |
| ANICB320-06 | BOLD:AAC0938 | Epicoma | *Epicoma anisozyga* | species | AU | Australasia |
| ANICD091-10 | BOLD:AAC0939 | Epicoma | *Epicoma anisozyga* | species | AU | Australasia |
| ANICD048-10 | BOLD:AAO5991 | Epicoma | *Epicoma argentata* | species | AU | Australasia |
| GWORV267-10 | BOLD:AAF0720 | Epicoma | *Epicoma argentata* | species | AU | Australasia |

| | | | | | | |
|---|---|---|---|---|---|---|
| ANICD046-10 | BOLD:ACE6810 | Epicoma | *Epicoma argentosa* | species | AU | Australasia |
| ANICD047-10 | BOLD:AAL0226 | Epicoma | *Epicoma argentosa* | species | AU | Australasia |
| GWORA147-08 | BOLD:AAB8419 | Epicoma | *Epicoma argentosa* | species | AU | Australasia |
| ANICD061-10 | BOLD:AAJ0572 | Epicoma | *Epicoma asbolina* | species | AU | Australasia |
| GWORA240-08 | BOLD:AAJ0573 | Epicoma | *Epicoma asbolina* | species | AU | Australasia |
| ANICD066-10 | BOLD:AAN3262 | Epicoma | *Epicoma barnardi* | species | AU | Australasia |
| ANICD052-10 | BOLD:AAJ0581 | Epicoma | *Epicoma barytima* | species | AU | Australasia |
| ANICD053-10 | BOLD:AAO7337 | Epicoma | *Epicoma barytima* | species | AU | Australasia |
| WALPA2354-12 | BOLD:AAO7336 | Epicoma | *Epicoma barytima* | species | AU | Australasia |
| ANICD054-10 | BOLD:AAO1162 | Epicoma | *Epicoma chrysosema* | species | AU | Australasia |
| AMUB438-11 | BOLD:AAE4068 | Epicoma | *Epicoma contristis* | species | AU | Australasia |
| ANICD096-10 | BOLD:AAD8944 | Epicoma | *Epicoma contristis* | species | AU | Australasia |
| GWORV389-10 | BOLD:AAJ0574 | Epicoma | *Epicoma derbyana* | species | AU | Australasia |
| ANICD073-10 | BOLD:AAE4056 | Epicoma | *Epicoma dispar* | species | AU | Australasia |
| ANICD074-10 | BOLD:ABX4947 | Epicoma | *Epicoma dispar* | species | AU | Australasia |
| AMUB464-11 | BOLD:AAA8041 | Epicoma | *Epicoma melanospila* | species | AU | Australasia |
| ANICD077-10 | BOLD:AAA8041 | Epicoma | *Epicoma melanospila* | species | AU | Australasia |
| LOTSA340-06 | BOLD:AAD1424 | Epicoma | *Epicoma melanospila* | species | AU | Australasia |
| ASMII13020-22 | BOLD:AAM3010 | Epicoma | *Epicoma melanosticta* | species | AU | Australasia |
| IMLQ466-08 | BOLD:AAE3998 | Epicoma | *Epicoma melanosticta* | species | AU | Australasia |
| ANICD058-10 | BOLD:AAJ0564 | Epicoma | *Epicoma phoenura* | species | AU | Australasia |
| ANICD095-10 | BOLD:ACE7103 | Epicoma | *Epicoma pontificalis* | species | AU | Australasia |
| ANIC732-06 | BOLD:AAD9054 | Epicoma | *Epicoma protrahens* | species | AU | Australasia |
| ANICD102-10 | BOLD:ACF5969 | Epicoma | *Epicoma protrahens* | species | AU | Australasia |
| NSWHO5509-18 | BOLD:ADT5732 | Epicoma | *Epicoma protrahens* | species | AU | Australasia |
| ANICD083-10 | BOLD:AAN9684 | Epicoma | *Epicoma signata* | species | AU | Australasia |
| ANICD087-10 | BOLD:AAN3062 | Epicoma | *Epicoma signata* | species | AU | Australasia |
| ANICD044-10 | BOLD:AAF0720 | Epicoma | *Epicoma subargentea* | species | AU | Australasia |
| ANICD086-10 | BOLD:AAD8944 | Epicoma | *Epicoma zelotes* | species | AU | Australasia |
| MAMOT1751-12 | BOLD:AAZ0788 | Gazalina | *Gazalina apsara* | species | PK | Indomalayan |
| BHULP620-22 | BOLD:AEP3838 | Gazalina | *Gazalina chrysolopha* | species | BT | Indomalayan |

| | | | | | | |
|---|---|---|---|---|---|---|
| MAMOT1758-12 | BOLD:AAN5625 | Gazalina | *Gazalina chrysolopha* | species | PK | Indomalayan |
| CWLMA199-15 | BOLD:ACX7855 | Hypsoides | *Hypsoides* sp. | genus | MG | Afrotropic |
| ANICD107-10 | BOLD:AAN9608 | Mesodrepta | *Mesodrepta harpotoma* | species | AU | Australasia |
| ANIC499-06 | BOLD:AAB0313 | Ochrogaster | *Ochrogaster lunifer* | species | AU | Australasia |
| ANICD027-10 | BOLD:AAB0319 | Ochrogaster | *Ochrogaster lunifer* | species | AU | Australasia |
| ANICD028-10 | BOLD:AAB0315 | Ochrogaster | *Ochrogaster lunifer* | species | AU | Australasia |
| ANICD033-10 | BOLD:AAB0318 | Ochrogaster | *Ochrogaster lunifer* | species | AU | Australasia |
| GBGL38573-19 | BOLD:ADR7035 | Paradrallia | *Paradrallia rhodesi* | species | ZM | Afrotropic |
| ANICD039-10 | BOLD:AAN9487 | Tanystola | *Tanystola isabella* | species | AU | Australasia |
| BIBSA1730-16 | BOLD:AAK2887 | Thaumatopoea | *Thaumatopoea pityocampa* | species | IT | Palearctic |
| LTOLB930-11 | BOLD:ACF4875 | Thaumetopoea | *Thaumetopoea abyssinica* | species | KE | Afrotropic |
| GBGL12179-13 | BOLD:ACH8435 | Thaumetopoea | *Thaumetopoea aff. pityocampa* | species | | Palearctic |
| GBGL38574-19 | BOLD:AAZ8816 | Thaumetopoea | *Thaumetopoea apologetica* | species | TZ | Afrotropic |
| LBEOA1147-11 | BOLD:AAW6612 | Thaumetopoea | *Thaumetopoea apologetica* | species | UG | Afrotropic |
| LBEOA1149-11 | BOLD:ACF4875 | Thaumetopoea | *Thaumetopoea apologetica* | species | KE | Afrotropic |
| GBGL12185-13 | BOLD:ACH8434 | Thaumetopoea | *Thaumetopoea bonjeani* | species | | Palearctic |
| BSNTN1587-24 | BOLD:AGA8844 | Thaumetopoea | *Thaumetopoea cretensis* | species | GR | Palearctic |
| LPALE1306-23 | BOLD:AEU3784 | Thaumetopoea | *Thaumetopoea cretensis* | species | GR | Palearctic |
| LBEOA1145-11 | BOLD:AAZ8814 | Thaumetopoea | *Thaumetopoea dhofarensis* | species | OM | Afrotropic |
| BCLEP011-16 | BOLD:ADB8365 | Thaumetopoea | *Thaumetopoea hellenica* | species | GR | Palearctic |
| GBGL12184-13 | BOLD:ACI2198 | Thaumetopoea | *Thaumetopoea herculeana* | species | | Palearctic |
| IBLAO1604-20 | BOLD:AEF8621 | Thaumetopoea | *Thaumetopoea herculeana* | species | ES | Palearctic |
| GBGL12183-13 | BOLD:ACH8432 | Thaumetopoea | *Thaumetopoea ispartaensis* | species | | Palearctic |

| | | | | | | |
|---|---|---|---|---|---|---|
| ANLMN4653-21 | BOLD:AEK2424 | Thaumetopoea | *Thaumetopoea latinivea* | species | MZ | Afrotropic |
| GBGL12182-13 | BOLD:ACH8433 | Thaumetopoea | *Thaumetopoea libanotica* | species | | Palearctic |
| BCLEP001-16 | BOLD:ACH8435 | Thaumetopoea | *Thaumetopoea mediterranea* | species | IT | Palearctic |
| GBGL12181-13 | BOLD:ACG4893 | Thaumetopoea | *Thaumetopoea pinivora* | species | | Palearctic |
| GBGL12180-13 | BOLD:AAK2887 | Thaumetopoea | *Thaumetopoea pityocampa* | species | | Palearctic |
| GBMNF23004-22 | BOLD:ADT6396 | Thaumetopoea | *Thaumetopoea pityocampa* | species | PT | Palearctic |
| ABOLA667-14 | BOLD:ABV0114 | Thaumetopoea | *Thaumetopoea processionea* | species | AT | Palearctic |
| GBGL12177-13 | BOLD:ACH5418 | Thaumetopoea | *Thaumetopoea solitaria* | species | | Palearctic |
| GBGL12176-13 | BOLD:ACI1213 | Thaumetopoea | *Thaumetopoea wilkinsoni* | species | | Palearctic |
| GBMNF23046-22 | BOLD:AGA8844 | Thaumetopoea | *Thaumetopoea wilkinsoni* | species | GR | Palearctic |
| GBMNF23058-22 | BOLD:AGA8844 | Thaumetopoea | *Thaumetopoea wilkinsoni* | species | GR | Palearctic |
| LECYP314-23 | BOLD:ACI1213 | Thaumetopoea | *Thaumetopoea wilkinsoni* | species | CY | Palearctic |
| GWOSZ657-11 | BOLD:ABV3081 | Thiacides | *Thiacides* sp. | genus | GH | Afrotropic |
| LBEOA1141-11 | BOLD:AAM9072 | Thiacides | *Thiacides* sp. | genus | SL | Afrotropic |
| LBEOA1143-11 | BOLD:AAM9072 | Thiacides | *Thiacides* sp. | genus | SL | Afrotropic |
| ANICD116-10 | BOLD:AAC0915 | Trichiocercus | *Trichiocercus mesomelas* | species | AU | Australasia |
| GWORW600-10 | BOLD:AAD6216 | Trichiocercus | *Trichiocercus mesomelas* | species | AU | Australasia |
| AMUB025-11 | BOLD:AAB7774 | Trichiocercus | *Trichiocercus sparshalli* | species | AU | Australasia |
| LOQT1055-07 | BOLD:AAJ8692 | Trichiocercus | *Trichiocercus sparshalli* | species | AU | Australasia |
| ANICD119-10 | BOLD:AAO0588 | Trichiocercus | *Trichiocercus celaena* | species | AU | Australasia |
| BHULP621-22 | BOLD:AEP3839 | Gazalina | *Gazalina transversa* | species | BT | Indomalayan |
| GBMIN85171-17 | BOLD:ADK2662 | Hypsoides | *Hypsoides antsianakana* | species | MG | Afrotropic |
| SAMPA132-08 | BOLD:AAG1464 | Hypsoides | *Hypsoides conglomerata* | species | MG | Afrotropic |
