# Supplementary figures and images for: Armoured Amazon female moths: urticating setae in Notodontidae (Lepidoptera)
Source: J Insect Sci. 2026 Jul 2;26(4):ieag051. doi: 10.1093/jisesa/ieag051 (PMC13326758; doi:10.1093/jisesa/ieag051)

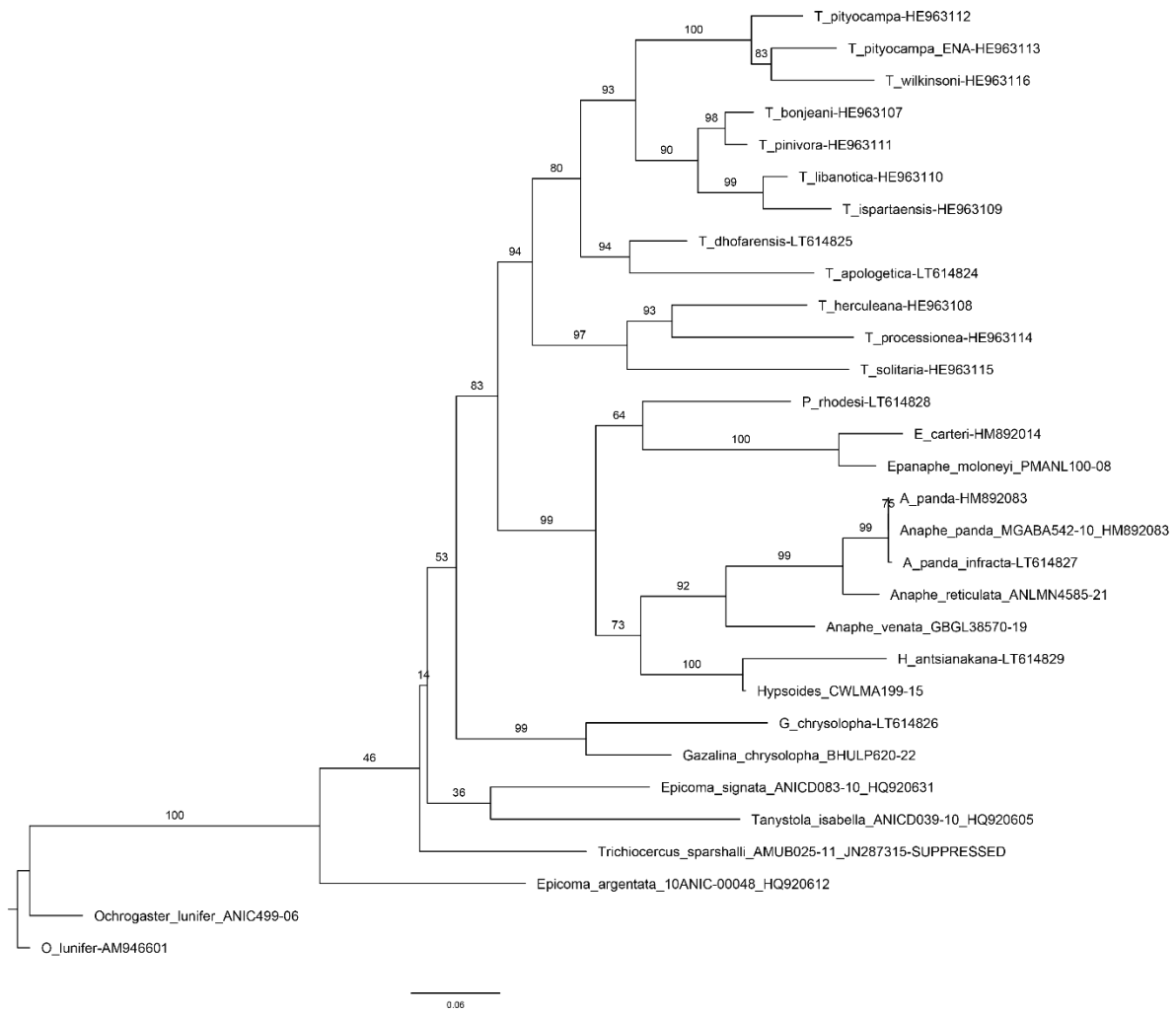

**Figure S3.** Notodontidae phylogenetic tree with the bootstrap values.

Supplement: ieag051_Supplementary_Data [file ieag051_supplementary_data.zip › Supplementary Figure S3.pdf]
